# Investigating the Antiscale Magnetic Treatment Controversy: Insights from the Model Calcium Carbonate Scalant

**DOI:** 10.1038/s41598-024-82048-9

**Published:** 2025-01-27

**Authors:** M. ElMassalami, M. S. Teixeira, A. Elzubair

**Affiliations:** 1 Physics Institute, Federal University of Rio de Janeiro, Post Office Box 68528, Rio de Janeiro, 21945-972 Brazil; 2 Military Institute of Engineering, Praça General Tibúrcio 80, Urca, Rio de Janeiro, RJ 22290-270 Brazil

**Keywords:** Controversies in magnetic anti-scale treatment, Magnetic field effects on pH, microstructure, and conductivity of ${\text{CaCO}}_{3}$, Magnetic field effects on polymorphism of ${\text{CaCO}}_{3}$, Influence of magnetic contamination on the stages of scale formation of  ${\text{CaCO}}_{3}$, Effect of pH on calcium carbonate ccale formation $.$, Electronic properties and materials, Magnetic properties and materials, Applied physics, Ferromagnetism, Magnetic properties and materials

## Abstract

The antiscale magnetic treatment (ASMT) claims to utilize magnetic field to combat scaling. However, its underlying mechanism, effectiveness, and reliability remain controversial. To address these contentious aspects, we analyze the influence of a magnetic field on the different stages of typical scale formation, using ${\text{CaCO}}_{3}$ as a model scale. For simplification, we consider the working fluid, such as in domestic and industrial settings, as a homogeneous mixture of a supersaturated, multi-ionic solution and a suspension of neutral multiphase contaminants, a fraction of which is magnetic. We argue that the combined effects of pH variation and catalytic role of magnetic contaminants are crucial factors affecting the properties of the resultant scale. Based on these considerations, we clarify the controversy by showing that each side holds a valid piece of the overall picture of the ASMT process. Indeed, the two viewpoints on magnetic field’s influence on scaling can be explained along the following scenarios: (i) Within a non-contaminated, supersaturated solution, there is no significant field influence because, under typical laboratory conditions, the Lorentz force does not practically affect the scaling process. (ii) Within a high-pH, magnetically-contaminated, supersaturated solution, the field does have an influence: Here, gradient-force-driven agglomerated particulates can act as templates for heterogeneous nucleation and growth.

## Introduction

In many domestic and industrial environments, where large volumes of fluids are stored or transported, the working fluid is often a supersaturated aqueous solution containing, in addition, significant amounts of suspended, multi-phase, neutral contaminants^1^. This combination is referred to as a *supersaturated-contaminant-bearing solution*. Due to chemical driving forces, nucleation and growth processes within the ionic subsystem^2^ lead to substantial deposits, commonly known as scale. When scaling becomes problematic, various mechanical, chemical, or physical methods are employed to combat it^1^. Ideally, an optimized anti-scaling strategy should be cost-effective, safe, environmentally friendly, and easy to implement, operate, and maintain without disrupting the process. One promising method is antiscale magnetic treatment (ASMT), which claims to use magnetic fields to reduce scaling^3–7^. However, ASMT has been the focus of ongoing debate concerning its underlying mechanisms, effectiveness, and reliability^5,6,8–21^.

In this work, our primary objective is to elucidate the contentious aspects surrounding this controversy by conducting a series of systematic laboratory experiments that monitor the response of a synthetic working fluid when subjected to a magnetic field. During these experiments, we performed in-situ, real-time monitoring of ionic concentration, ion mobility, and protonic variation. Additionally, we conducted ex-situ analyses to probe the elemental, structural, magnetic, polymorphic, and morphological properties of the resulting residues. These complementary analyses, particularly the magnetic studies, enabled us to identify and evaluate the influence of magnetic fields on the various stages of scaling. This comprehensive approach allowed us to unravel and address the contentious aspects of the ASMT controversy.

**Fig. 1:**
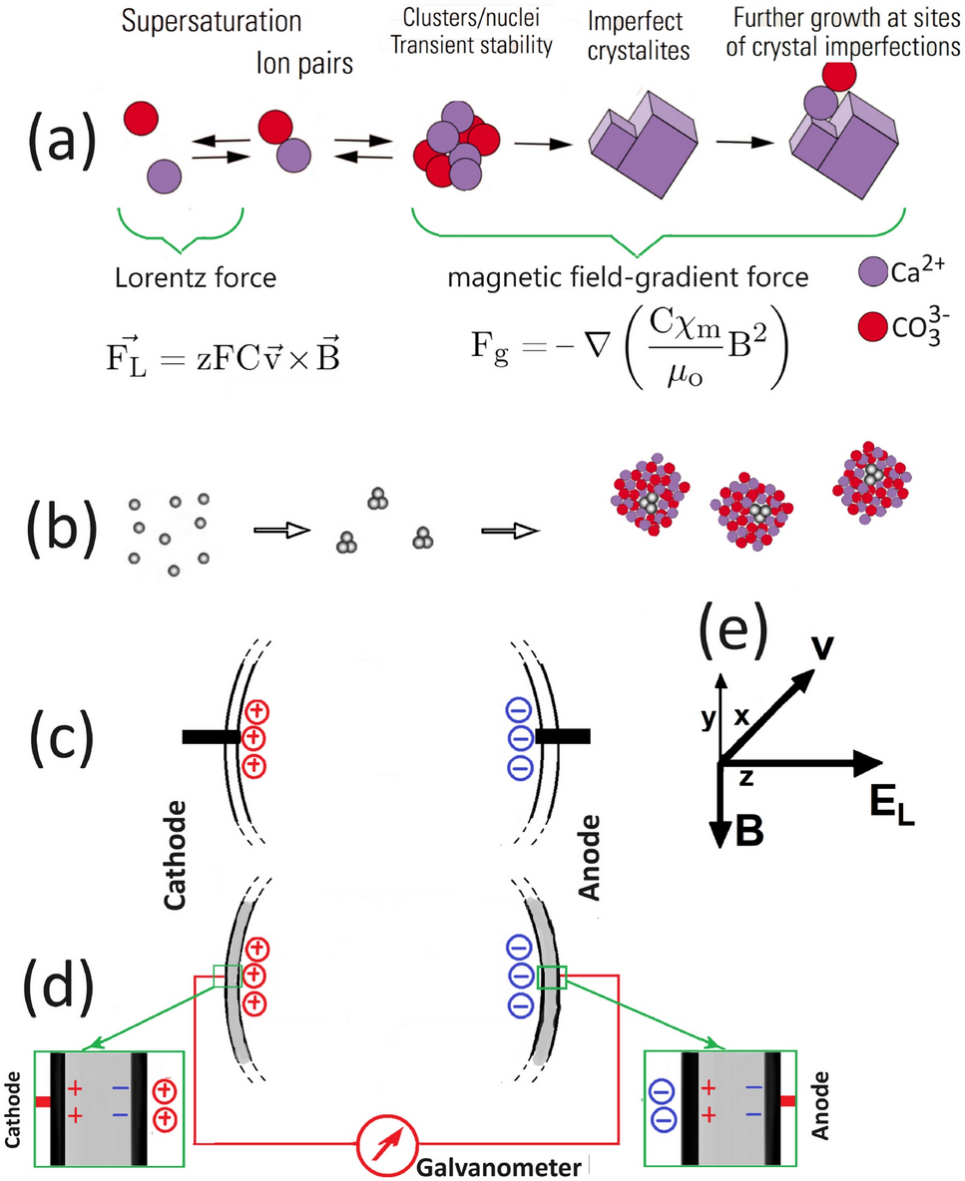
Schematics illustration of the influence of an applied magnetic field on the different stages of the nucleation and growth within a moving *supersaturated-contaminants-bearing solution*. (**a**) The nucleation and growth (adapted from Ref. ^1^) is roughly divided into two stages: (i) Before critical nucleation, ions are moving relative to field and are influenced by the Lorentz force as described in Eq. (1). (ii) The nucleated or grown neutral entities, having negative or positive susceptibilities, can be influenced by field-gradient force as in Eq. (2). (**b**) In the case of suspended ferromagnetic-like nano-contaminants, a gradient force would induce agglomeration, forming relatively large-sized particulates that can act as templates for heterogeneous nucleation and growth. We envisage a type of core-shell configuration, with a core of magnetic contaminant being encircled by a shell of diamagnetic matrix. We expect a distribution in dimensions of both core and shell. (**c**) Representation of the induced Faraday-Hall EMF arising at the internal boundaries of an insulating cell housing (only part of the cylinder cross-section is shown). Electrodes are represented by the short black bars and denoted as $\text{V}_1$ and $\text{V}_2$ in Fig. 2b. (**d**) The induced EMF at the cathodic and anodic regions of a cell with metallic housing. The green rectangle boxes highlight and expand the two regions wherein the Lorentz-force induced charge distribution leads to a surge of an induced current through the metallic case. The current is schematically represented as an *external* thin red line^5,9^. (**e**) Representation of *xyz* axes, right-hand rule for $\vec {{\text{B}}}$, average velocity $\vec {\text{v}}$, and induced electric field $\vec {\mathrm{E_{L}}}$ due to the accumulation of charges at the opposite boundaries.

An essential step in our analysis is identifying the forces that emerge when an external magnetic field is applied to a moving *idealized* working fluid which, as illustrated in Fig. 1a,b, is conceptualized as a homogeneous mixture of two subsystems, each involving the same nonmagnetic and incompressible solvent or carrier liquid.

The first subsystem consists of a supersaturated solution with multi-ionic components. If we assume that the ions exhibit weaker paramagnetic or diamagnetic properties, applying a magnetic field to such a solution, while it moves through an insulator-housing cell, induces a Lorentz force. For an i-th component with molar concentration ${{\text{C}}}_{\text{i}}$ and charge number ${\text{z}}_{\text{i}}$, the force density is given by^22–25^:

1 $$\begin{aligned} \vec {\mathrm{_i F_L}} = {\text{z}}_{\text{i}} {{\text{C}}}_{\text{i}} {{\text{F}}} \vec {\text{v}} \times \vec {{\text{B}}}, \end{aligned}$$

where ${{\text{F}}}$ is the Faraday constant, $\vec {\text{v}}$ is the average velocity, and $\vec {{\text{B}}}$ is the magnetic field (recalling ${\text{B}} = {\text{H}} + 4\pi {\text{m}}$, then since ${\text{m}} \approx 0$, ${\text{B}} \approx {\text{H}}$). The net effect is an induced electromotive force (EMF) which is independent of concentration and type of the ionic subsystem^26^ and, moreover, is accompanied by an electric force that balances the Lorentz force^9,22,23,27–32^. Calculations based on Eq. (1) indicate that applying a field to a moving ionic fluid causes minor alterations in the local ionic concentration at the boundary layers of the cell, as shown in Fig. 1c,d. These localized ionic modifications affect the local activity product and, consequently, the local chemical potential difference, which is the driving force behind nucleation and growth processes^2^. However, under normal laboratory conditions, these field-induced modifications are independent of the chemical nature of the local ionic concentration and are typically within a micromolar range^4,26^. Therefore, while these changes may produce a measurable Hall voltage, they do not have a practical impact on the scaling process.

The second subsystem contains all contaminating neutral, multiphase entities, which can be either diamagnetic, paramagnetic, or ferromagnetic-like particulates. When this subsystem is subjected to a gradient in field $(\nabla {\text{B}})$ or in magnetic susceptibility $(\nabla ({\text{C}}\chi _{\text{m}}))$, while keeping all other control parameters constant, a force density arises^24,33,34^:

2a $$\begin{aligned} \vec {\mathrm{F_{1}}} =&-\nabla \left( \frac{{\text{C}}\chi _{{\text{m}}}}{2\mu _\text{o}}{\text{B}}^2\right) , \quad \text {( the general case)} \end{aligned}$$

2b $$\begin{aligned} \vec {\mathrm{F_{2} }}=&-\frac{{\text{C}}\chi _{{\text{m}}}{\text{B}} \nabla {{\text{B}}}}{\mu _\text{o}}, \quad \quad \text {(where } \nabla {\text{B}} \ne 0; \nabla \chi _{\text{m}} = \nabla {\text{C}}=0\text {)} \end{aligned}$$

2c $$\begin{aligned} \vec {\mathrm{F_{3}}} =&-\frac{{\text{B}}^2 \nabla ({\text{C}}\chi _{{\text{m}}})}{2\mu _\text{o}}, \quad \text {(where } \nabla ({\text{C}}\chi _{\text{m}}) \ne 0; \nabla {\text{B}}=0\text {)}. \end{aligned}$$

here $\mu _\text{o}$ is medium permeability, $\chi _{\text{m}}$ the molar susceptibility, and ${\text{C}}$ the molar concentration. Although a uniform $\vec {{\text{B}}}$ exerts no action on uniformly-distributed neutral magnetic components^24,35^, a gradient-related force, as described in Eq. (2), drives various processes. These include magnetic-induced polymorphic selectivity^36^, field-induced modification of crystal growth^36–39^, magnetic levitation^40–44^, magnetophoresis-type processing^45–48^, chirality selection^49^, magneto-Archimedes separation^50^, and liquid crystal alignment^51^.

Most of the gradient forces mentioned above, due to their limited range and specific conditions for applicability, have not been widely used in ASMT. In applications like water treatment, desalination, and oil and gas extraction, the atomic constituents of the most common scalants (such as ${\text{CaCO}}_{3}$, ${\text{BaSO}_4}$, or ${\text{SrSO}_4}$) are diamagnetic, resulting in relatively weak forces. However, the presence of ferromagnetic-like contaminants, such as oxides of iron, manganese, or chromium, significantly changes this scenario. These contaminants often have high Curie points (${\text{T}}_{\text{C}}$) and may be released into the working fluid from iron-based components used in storage tanks, control units, hydraulic pumps, or metallic tubing. When subjected to a strong magnetic field gradient, these ferromagnetic-like particulates tend to migrate and agglomerate in regions of high magnetic field strength (or regions of low field strength, in the case of diamagnetic materials)^24,25^. These field-induced aggregates can act as heterogeneous templates for nucleation and growth. The process whereby nanosized scale precipitates in the fluid bulk without adhering to the inner surfaces of the tubing^52^ is often celebrated as a magnetically-driven reduction of scaling. It is worth emphasizing that this additional scaling channel operates across the whole magnetically-active volume and competes with the traditional surface-related scaling channel which independently operates over a thin sheet of supersaturated solution within the immediate neighbourhood of the defective surface. Worth noting, the above-mentioned mechanism of magnetically-induced entrainment of nanosized scale within the bulk explains the previously reported colloidal stability, eliminating the need for an explanation based on the formation of hydrophilic crystallites with modified surface charge^5^.

The above-mentioned suspension of ferromagnetic nanoparticles in a supersaturated solution is reminiscent of a ferrofluid-a suspension of magnetic nanoparticles in an appropriate carrier liquid^53,54^. However, there are stark differences. In the present case, the carrier liquid is a supersaturated solution, and the ferromagnetic particles are extremely diluted, uncoated, surfactant-less, and lack interparticle repulsive forces, distinguishing them significantly from traditional ferrofluids (see also Supplementary Materials Section “SM3”). Moreover, the focus here is not on the rheological properties of the carrier fluid but rather on the gradient-induced agglomeration of individual particles and how this influences the kinetics and stability of the deposition from the supersaturated solution.

One of the most commonly used technique for studying the magnetic field’s impact on the scale formation is the ex-situ structural analysis. ${\text{CaCO}}_{3}$ is particularly suitable for studying how field impacts each of its different microstructure forms, including polymorphism and morphologies^52,55–58^. ${\text{CaCO}}_{3}$ has three main anhydrous phases: calcite, aragonite, and vaterite. Calcite is the most common and thermodynamically stable phase, forming under a wide range of conditions. Aragonite is less stable and less common, while vaterite is the least stable and often transforms into calcite or aragonite over time. These less-stable forms can be selectively stabilized and preserved by specific foreign agents. For example, in vaterite pearls or vaterite minerals, organic additives or templates such as proteins and polysaccharides are essential for stabilization. In contrast, aragonite pearls are stabilized by the presence of magnesium ions and other environmental factors^59^. One of the main findings of this work is that magnetic contaminants, acting as a foreign agent and being susceptible to field influence, can indirectly allow an applied field to selectively stabilize and preserve one or the other of these less stable forms.

As mentioned above, all anhydrous ${\text{CaCO}}_{3}$ polymorphs are diamagnetic with similar susceptibility values. Consequently, it is challenging to use these susceptibility differences, along with the forces described in Eq. (2), to induce changes in the polymorphic content under typical laboratory conditions. Instead, modifying the kinetics of nucleation and growth during scaling is more feasible by varying control parameters such as temperature, pressure, contaminants, pH, or the concentrations of ${\text{Ca}}^{2+}$ and ${\text{CO}}_3^{2-}$^52^. In this work, the most influential factors are pH level and type and content of magnetic impurities.

The impact of impurities, especially magnetic ones, was briefly discussed earlier. The effect of pH on ${\text{CaCO}}_{3}$ formation can be best understood using the Bjerrum plot of the carbonate system^60,61^. At higher pH levels, the concentration of ${\text{CO}}_3^{2-}$ increases, leading to faster nucleation and greater stability of ${\text{CaCO}}_{3}$. In contrast, lowering the pH shifts the equilibrium towards $\text{CO}_{2}$ and ${\text{HCO}}_{3}^{-}$, reducing the ${\text{CO}}_3^{2-}$ concentration. This results in a longer induction time and decreased stability, potentially causing the dissolution of pre-existing ${\text{CaCO}}_{3}$ polymorphs.

For better clarity on pH-related considerations, let us analyze two reactions leading to supersaturation of ${\text{CaCO}}_{3}$. The first, the bicarbonate route, involves:

3 $$\begin{aligned} {{\text{CaCl}}_{\mathrm{2(aq)}}} + 2{\text{NaHCO}_{\mathrm{3(aq)}}} \rightarrow {\text{CaCO}_{\mathrm{3(s)}}} + 2{\text{NaCl}_{\mathrm{(aq)}}} + {\text{CO}_{\mathrm{2(g)}}} + {{\text{H}}_{2}\text{O}_{\mathrm{(l)}}}, \end{aligned}$$

The second reactions, the carbonate route, is described by:

4 $$\begin{aligned} {{\text{CaCl}}_{\mathrm{2(aq)}}} + {\text{Na}_{2} \text{CO}_{\mathrm{3(aq)}}} \rightarrow {\text{CaCO}_{\mathrm{3(s)}}} + 2{\text{NaCl}}_{\mathrm{(aq)}}. \end{aligned}$$

Empirically, the initial pH for reactions involving Eqs. (3, 4) is relatively high, approximately 8.5 and 11.5, respectively. Here, the progress of these reactions are chosen to proceed by an incremental addition of ${{\text{CaCl}}_{\mathrm{2(aq)}}}$ (see Methods). Such a procedure leads to a distinct pH evolution: For the bicarbonate route (Eq. 3), $\text{CO}_{2}$ is produced, which on dissolving into water,

5a $$\begin{aligned}&{\text{CO}_{\mathrm{2(g)}} \leftrightarrow \text{CO}_{\mathrm{2(aq)}}}, \end{aligned}$$

5b $$\begin{aligned}&{\text{CO}_{\mathrm{2(aq)}} + \text{H}_{2}\text{O} \leftrightarrow {\text{H}}^{+} + \text{HCO}^{-}_{3}}, \end{aligned}$$

5c $$\begin{aligned}&{\text{HCO}_{3}^{-} \leftrightarrow {\text{H}}^{+} + \text{CO}^{2-}_{3}}, \end{aligned}$$

leads to a drop in pH; thus nucleation and growth processes related to Eq. (3) occur at relatively low pH levels (from 8.5 down to around 7). In contrast, reactions (nucleaton and growth) related to Eq. (4) start and are maintained under higher pH conditions; though dropping from approximately 11.5, still it is above 8.5. As will be shown below, the regulatory role of pH environment, combined with the scenario of magnetic contaminants acting as templates, allows for a field-enabled manipulation of the scale properties. We show below that this understanding clarifies many of the contentious issues central to ASMT controversy.

The format of this paper is outlined as follows: In Section “Methods”, we detail the experimental setups and methodologies used. Some useful remarks on each of the employed analytical techniques are provided in §SM1-SM4. The main findings are reported in Section “Results and analysis” (also in Supplementary Materials, SM), where they are organized and analyzed according to the measured physical properties. This section emphasizes the evolution of ionic concentration and mobility, proton concentration, microstructure, and magnetic properties as any specific control parameter is varied. In Sections “Results and analysis” to “Discussion”, we discuss the factors driving this evolution, particularly focusing on the field-contamination-driven microstructure-shaping effect. Finally, summaries and conclusions, including an analysis of the ASMT , are presented in Section “Summary and conclusions”.

**Fig. 2:**
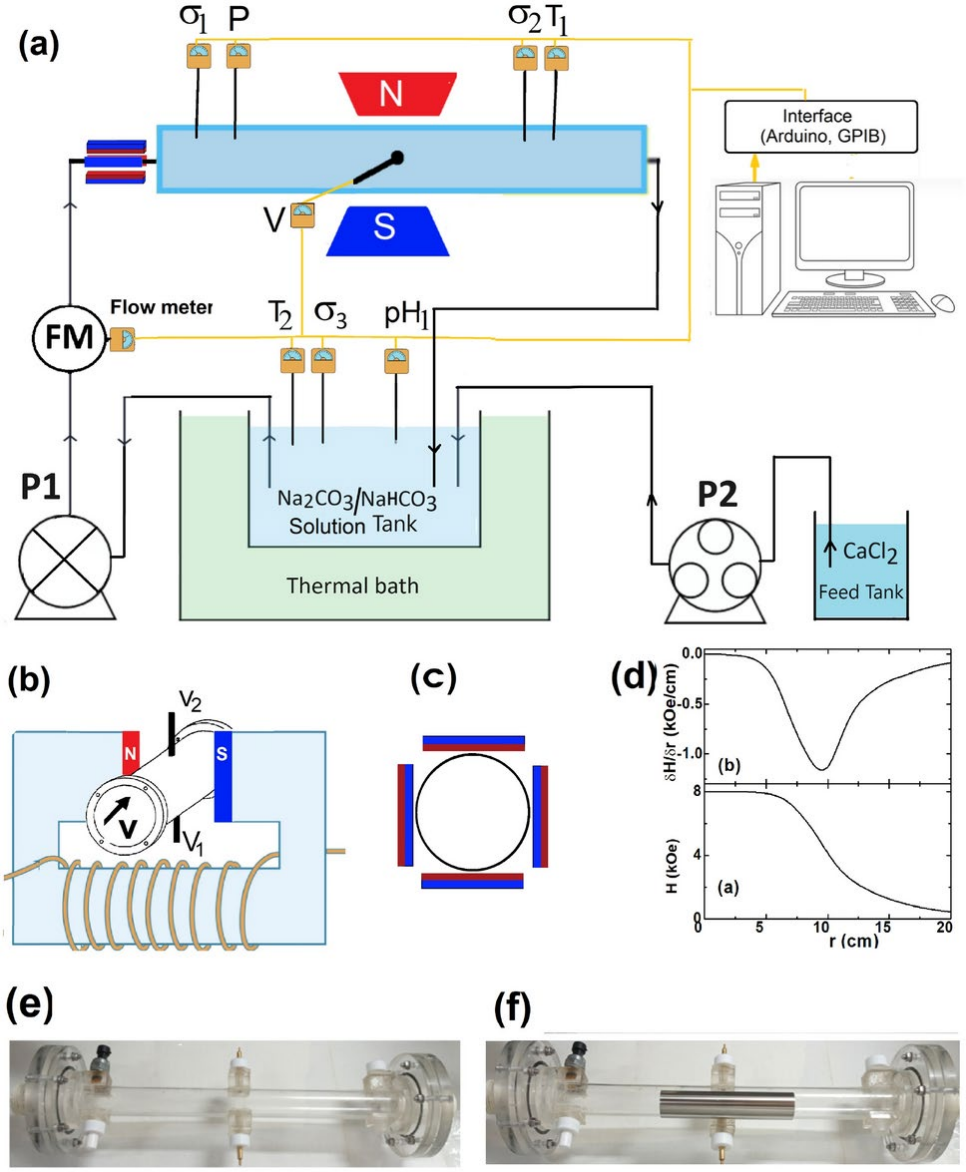
The experimental setup. (**a**) Schematic of the setup showing the hydraulic, control, sensor, and interface and acquisition subsystems. The cell (light-blue rectangle) is connected through flexible plastic tubing to the Solution Tank, which is in thermal contact with the Thermal bath tank. A solution of ${\text{NaHCO}_3}$ or ${\text{Na2CO}_{3}}$ is circulated by a centrifugal pump, ${\text{P}}_1$. ${\text{CaCl}}_{2\mathrm{(aq)}}$ is added using a peristaltic pump, ${\text{P}}_2$. Various probes are positioned either within the cell, along the pipeline, or within the solution tank: conductivity ($\sigma _\text{i}$), pH ($\text{pH}_\text{i}$), temperature (${\text{T}}_\text{i}$), manometer (P), flow meter (FM), and a pair of electrodes (Cu-Cu, Stainless Steel-Stainless Steel (SS-SS), Al-Al, or Brass-Brass). We observed that energy losses associated with flow restriction and increased pressure drop—due to the tubing or the insertion of auxiliary probes—lead to an increase in the temperature of the main solution beyond the capacity of our thermal bath. However, this temperature increase, which is at most $18^\circ \text {C}$, was found to have little influence on the reaction kinetics. The electrode pairs (5 mm diameter, mounted in the middle of the cell with an approximate $\omega \approx 5$ cm separation) are linked to a 10-channel voltage scanner. All input and control signals are routed via an Arduino and GPIB interface to or from a desktop computer; through the latter, all automation and data processing are managed. (**b**) The cell is placed between the poles (10 cm in diameter and 6 cm apart) of the electromagnet with $|\vec {{\text{H}}}| \le 10$ kOe. This arrangement allows large liquid volume to be exposed to higher magnetic dose^5^. (**c**) Schematic cross-section illustrating the arrangement of four bar magnets positioned around a cylinder with a 5.0 cm diameter. (**d.a**) The field distribution along the cell axis, the *x-axis*, with the origin being at the center. (**d.b**) The field gradient manifesting an extremum around 9.5 cm away from the center. (**e**) Photograph of the non-conductive transparent polycarbonate cylinder. (**f**) Photograph of the cell with a stainless steel cylinder, all fit within the electromagnet poles, within the homogeneous part of the magnetic field (see *panel b*).

**Fig. 3:**
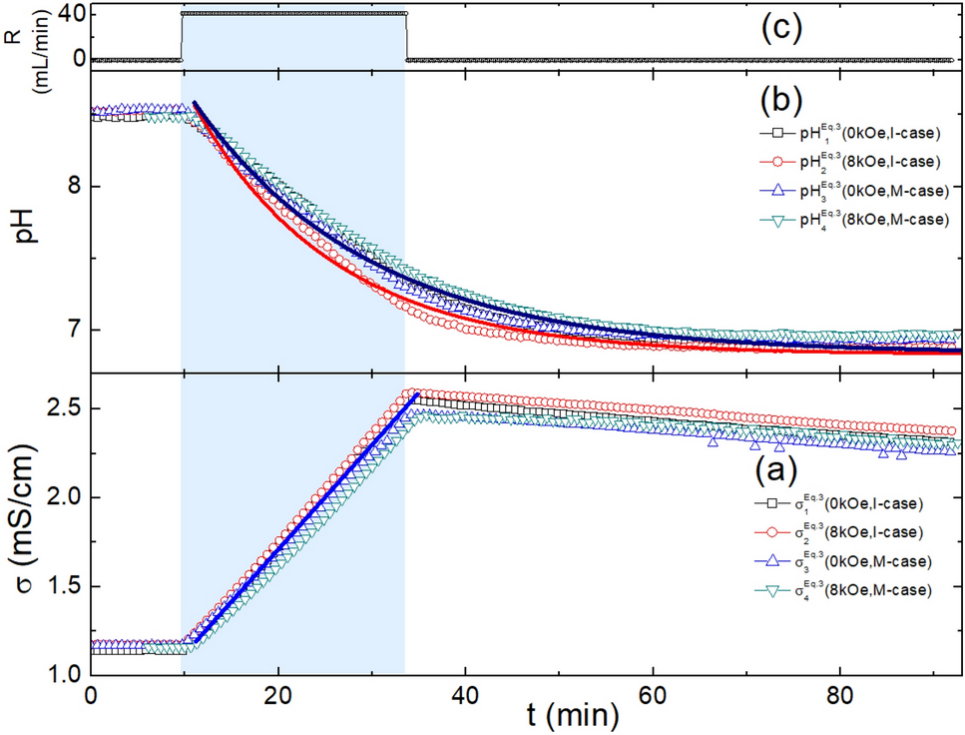
Evolution of the conductivity and pH, monitoring the reaction of Eq. (3) when subjected to a variation in H or Case. (**a**) Evolution of $\sigma ^{\mathrm{Eq.3}}_{\text{i} }$(H, Case) (i = 1 to 4, H = 0.0, 8.0kOe. Case = I-case, M-case). The thick blue line emphasizes the linear evolution of $\sigma ^{\mathrm{Eq.3}}_{\text{i} }$(H, Case, i = 1 to 4), (see text). (**b**) Evolution of $\text{pH} ^{\mathrm{Eq.3}}_{\text{i} }$(H, Case) (i = 1 to 4; H = 0.0, 8.0kOe; Case = I-case, M-case). The solid curves simulate the exponentially decaying function of Eq. (7) which starts directly after introducing ${\text{CaCl}}_{2\mathrm{(aq)}}$. (**c**) Evolution of the rate of ${\text{CaCl}}_{2\mathrm{(aq)}}$ addition to the circulating $\text{NaHCO}_{\mathrm{3(aq)}}$ solution. The light-blue area highlights the onset and duration of adding ${\text{CaCl}}_{2\mathrm{(aq)}}$ till complete neutralization of ${\text{NaHCO}_3}$.

**Fig. 4:**
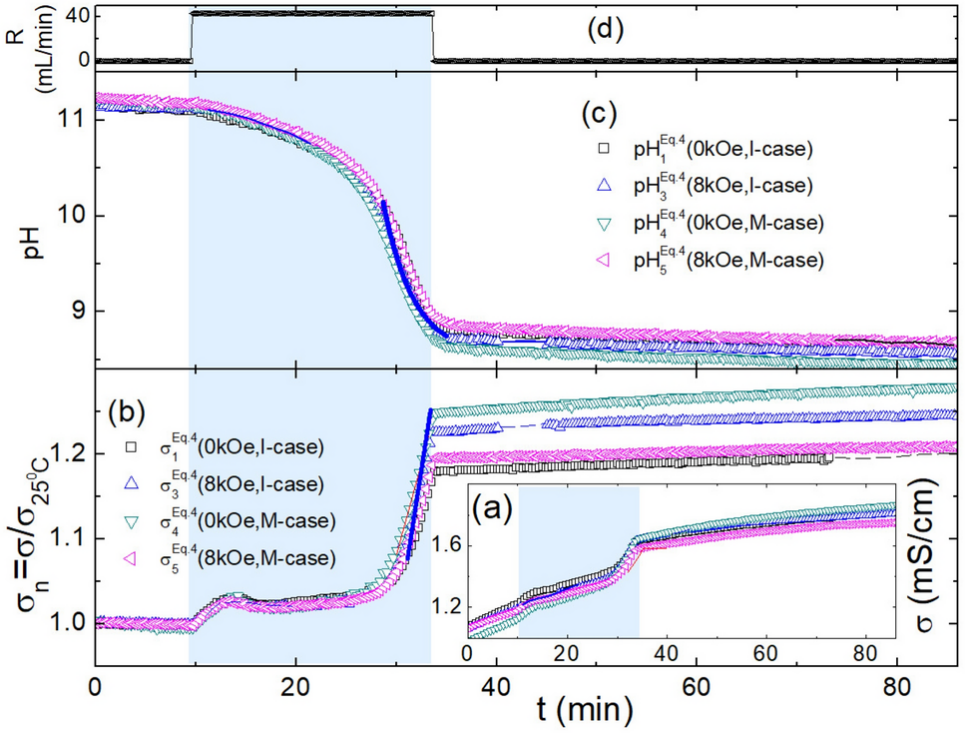
Evolution of conductivity and pH, monitoring the reaction of Eq. (4) when subjected to a variation in H or Case. (**a**) Evolution of $\sigma ^{\mathrm{Eq.4}}_{\text{i} }$(H, Case) (i = 1 to 4, H = 0.0, 8.0kOe,Case = I-case, M-case). Within the range preceding the addition of ${\text{CaCl}}_{2\mathrm{(aq)}}$, $\sigma$ must be constant. Accordingly, the observed linear-in-time variation must be related to a linear-in-temperature evolution, driven by the Joule conversion of the mechanical work of P1 pump (see Fig. 2). On subtracting this thermal effect and normalizing to $\sigma _{25 ^{\circ }{\text{C}}} \equiv \sigma ^{\mathrm{Eq.4}}_{\text{i} }\mathrm{(H, Case, 25}^{\circ }\mathrm{C)}$, we obtained (**b**) the evolution of $\sigma ^{\mathrm{Eq.4}}_{\text{i} }$(H, Case) (i = 1 to 4, H = 0.0, 8.0kOe, Case = I-case, M-case). The thick solid blue line emphasizes the linear rise of $\sigma ^{\mathrm{Eq.4}}_{\text{i} }$(H, Case) (see text). (**c**) Evolution of $\text{pH} ^{\mathrm{Eq.4}}_{\text{i} }$(H, Case) (i = 1 to 5, H = 0.0, 8.0kOe, Case = I-case, M-case). The thick solid blue curve simulates the exponential decay of $\text{pH} ^{\mathrm{Eq.4}}_{\text{i} }$(H, Case) as expressed in Eq. (7). **(d)** Evolution of the rate of the controlled addition of ${\text{CaCl}}_{2\mathrm{(aq)}}$ to the circulating $\text{Na}_{2}\text{CO}_{\mathrm{3(aq)}}$ solution.

**Table 1:**
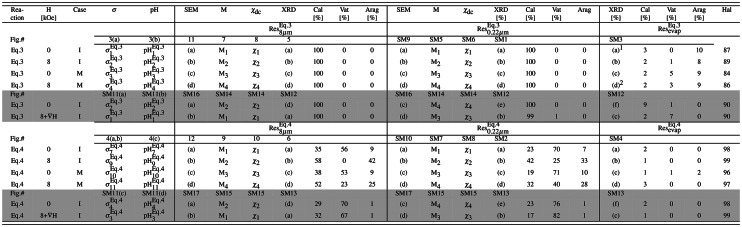
List of representative experiments conducted under specified conditions, including the type of reaction (described by Eq. (3) or Eq. (4), magnetic field (0, 8 kOe, or 8 kOe+$\nabla {\text{H}}$), cell casing (I: plastic insulating cylinder as shown in Fig. 2e, or M: metallic conducting cylinder as shown in Fig. 2f)), and content of contaminants (experiments with less contamination are indicated by Bold values; see Section “On the dependence of non-uniform field and contaminant content”).

For each experiment, we referenced the figure (Fig.#) containing curves of one of the associated two in-situ measurements or the corresponding four ex-situ measurements on each of ${\text {Res}}_{8\mu \text {m}}^{\text {Eq.3/Eq.4}}$, ${\text {Res}}_{0.22\mu \text {m}}^{\text {Eq.3/Eq.4}}$, or ${\text {Res}}_{\text {evap}}^{\text {Eq.3/Eq.4}}$. The in-situ measurements include conductivity ($\sigma ^{\mathrm{Eq.3/Eq.4}}_{\text{i}}$) and pH ($\text{pH}^{\mathrm{Eq.3/Eq.4}}_{\text{i}}$). The ex-situ measurements include microstructure (SEM), magnetization (${\text{M}}_{\text{i}}$), DC susceptibility ($\chi _{\text{i}}$), refined X-ray diffractogram (XRD), and the percentages of the polymorphic phases of calcite (Cal), vaterite (Vat), aragonite (Arg), and halite (Hal). The integers (i = 1, 2, $\ldots$) and letters (a,b,..) denote the curve or panel in the corresponding figure. We note the presence of unidentified contaminating peaks (much weaker than the minority phases) emerging in only a few cases, shown in the footnotes. The random manifestation of these peaks, with no correlation to the controlled parameters, does not influence the conclusions drawn in this work.

1 Unidentified weak peaks at *d* = 3.07, 2.63, 2.25 Å.

2 Unidentified weak peaks at *d* = 3.12, 2.96, 2.91, 2.62, 2.55, 2.26 Å

## Results and analysis

### Conductivity and pH analysis

#### Conductivity and pH monitoring of the reaction via ${\text{NaHCO}_{3}}$ route (Eq. 3)

Representative conductivity $\sigma ^{\mathrm{Eq.3}}_{\text{i}}$(H, Case) curves are shown in Fig. 3. The overall evolution of $\sigma ^{\mathrm{Eq.3}}_{\text{i}}$(H, Case) can be divided into three stages: (i) Before the addition of ${\text{CaCl}}_{2\mathrm{(aq)}}$, $\sigma ^{\mathrm{Eq.3}}_{\text{i}}$(H, Case) remains constant. Any variation is due to temperature changes (see caption of Fig. 2). (ii) During the controlled addition of ${\text{CaCl}}_{2\mathrm{(aq)}}$, $\sigma ^{\mathrm{Eq.3}}_{\text{i}}$(H, Case) increases linearly with a slope of 54.6(1) $\mu \text{S} \text {cm}^{-1} \text {min}^{-1}$. (iii) After completing the addition of ${\text{CaCl}}_{2\mathrm{(aq)}}$, $\sigma ^{\mathrm{Eq.3}}_{\text{i}}$(H, Case) shows a slight decreasing trend, occasionally with a weak hump superimposed. The slight decrease and the steady-state value of $\sigma ^{\mathrm{Eq.3}}_{\text{i}}$(H, Case) can be attributed to the combined effects of reduced ionic concentration, decreased ion mobility due to increased turbidity, and rising temperature.

The linear evolution of $\sigma ^{\mathrm{Eq.3}}_{\text{i}}$(H, Case) in Fig. 3 is a direct result of adding a constant, small quantity of ${\text{CaCl}}_{2\mathrm{(aq)}}$ (41.7 mL/min) to a large, approximately 13 L, solution. Based on the experimental arrangement and methodology of Fig. 2a, this statement can be used to simplify the determination of the rate law using the isolation method^62^. Here, the relatively abundant [$\text{NaHCO}_{3}$], compared to [${{\text{CaCl}}_{2}}$], is approximated as constant. One then obtains an overall pseudo-first-order rate law^62^ for the reaction in Eq. (3) which is, most probably, first-order in [${{\text{CaCl}}_{2}}$]:

6 $$\begin{aligned} \text {Rate} = {\text{K}}\times {\text{F}}([{\text{NaHCO}_{3}}])\times [{{\text{CaCl}}_{2}}] = {\text{K}}_1\times [{{\text{CaCl}}_{2}}], \end{aligned}$$

where ${\text{K}}$ is the overal rate constants and ${\text{K}}_1={\text{K}} \times [{\text{NaHCO}_{3}}]^2$, expressed as pseudo-second-order rate constant. According to Eq. (6), the constant injection of ${{\text{CaCl}}_{2}}$ leads to a precipitation of conductively-neutral ${\text{CaCO}}_{3}$ and a production of electrolytic NaCl within the light-blue area of Fig. 3: Thus the linear increase in $\sigma ^{\mathrm{Eq.3}}_{\text{i}}$(H, Case).

Representative $\text{pH} ^{\mathrm{Eq.3}}_{\text{i}}$(H, Case) curves in Fig. 3 show a monotonic decrease. The precipitation of ${\text{CaCO}}_{3}$ removes ${\text{CO}_3^{2-}}$, promoting the reaction ${\text{HCO}_{3}^{-} -> {\text{H}}^{+} + \text{CO}^{2-}_{3}}$, leading to a reduction in pH. SEM micrographs do not indicate strong dendritic morphology, suggesting that the reaction rate is the determining mechanism, and growth is expected to be exponential^57^. Fig. 3b shows an exponential decrease from 8.5 (typical of $\text{NaHCO}_{\mathrm{3(aq)}}$) to a nearly neutral steady-state value (typical of $\text{NaCl}_{\mathrm{(aq)}}$):

7 $$\begin{aligned} \text{pH} = \text{pH}_{\infty } + \text{A}_{\text{ph}} \text{exp} \left( \frac{-{\text{T}} + {\text{T}}_0}{\tau } \right) , \end{aligned}$$

where $\text{A}_{\text{ph}}$ measures the range of variation, $\tau$ is the time constant associated with the kinetics of reaction, and $\text{pH}_{\infty }$ is the steady-state value, which is influenced by water ionization and the production, absorption, or degassing of $\text{CO}_{2}$. Empirically, the parameters in Eq. (7) cluster around $\text{pH}_{\infty } \approx 6.84\sim 6.83$, $\text{A}_{\text{ph}} \approx 3.2 \sim 3.5$, and $\tau \approx 15\sim 19$ min.

The approximation of the evolution of conductivity as linear and that of pH as exponential is satisfactory but not perfect; as expected from the above argument, these approximations are corollaries of the pseudo-first-order rate law.

#### Conductivity and pH monitoring of the reaction via ${\text{Na}_{2}\text{CO}_{3}}$ route (Eq. 4)

The distinct chemical pathways of the reactions in Eqs. (3)–(4) are expected to lead to distinct features in the corresponding conductivities and pH. This expectation is confirmed by the following observations:

First, all $\text{pH} ^{\mathrm{Eq.4}}_{\text{i}}$(H, Case) values are higher than $\text{pH} ^{\mathrm{Eq.3}}_{\text{i}}$(H, Case), indicating a higher likelihood of forming vaterite and aragonite polymorphs. This is because the induction times for these polymorphs decrease as pH increases. In contrast, calcite has a relatively longer induction time and is less influenced by pH variations. During the ${\text{CaCl}}_{2\mathrm{(aq)}}$ addition, $\sigma ^{\mathrm{Eq.4}}_{\text{i}}$(H, Case) and $\text{pH} ^{\mathrm{Eq.4}}_{\text{i}}$(H, Case) in Fig. 4 exhibit a two-event process. Such a feature is not evident in $\sigma ^{\mathrm{Eq.3}}_{\text{i}}$(H, Case) and $\text{pH} ^{\mathrm{Eq.3}}_{\text{i}}$(H, Case) in Fig. 3, as a lower pH does not favor the formation of vaterite or aragonite. The precipitation of ${\text{CaCO}}_{3}$ begins with the formation of an unstable amorphous polymorph, the one with the least stability and highest solubility, which eventually transforms (if no stabilizing agent is introduced) into a more stable polymorph, calcite^52^. These multi-step transformations, which often occur at high pH, lead to the emergence of two events in $\sigma ^{\mathrm{Eq.4}}_{\text{i}}$(H, Case) and $\text{pH} ^{\mathrm{Eq.4}}_{\text{i}}$(H, Case) in Fig. 4: the first is attributed to the formation of vaterite/aragonite, and the second to the formation of calcite.

Second, after the initial increase of $\sigma ^{\mathrm{Eq.4}}_{\text{i}}$(H, Case) during the first few minutes of ${\text{CaCl}}_{2\mathrm{(aq)}}$ addition, an almost stationary state is reached where the conductivity remains nearly constant while $\text{pH} ^{\mathrm{Eq.4}}_{\text{i}}$(H, Case) weakly decreases. This evolution suggests that the rates of formation and dissolving of these non-calcite polymorphs are nearly equal. This state continues until approximately ${\text{T}} \approx 26$ minutes; thereafter, $\sigma ^{\mathrm{Eq.4}}_{\text{i}}$(H, Case) increases with a linear rate as described by Eq. (6), while $\text{pH} ^{\mathrm{Eq.4}}_{\text{i}}$(H, Case) decays exponentially as described by Eq. (7); as in Fig. 3, both features must be associated with the formation of calcite.

Third, the onset point, ${\text{T}}_{\text{onset}} \approx 26$ minutes, is considered a measure of the induction time of calcite (i.e., ${\text{T}}_{\text{ind}} \approx 16$ minutes), while the time constant in the exponential decay ($\tau \approx 3$ minutes) measures the rate of calcite formation. The time rate of the linearly evolving $\sigma ^{\mathrm{Eq.4}}_{\text{i}}$(t, H, Case) is around 50 $\mu \text{S}\text {cm}^{-1} \text {min}^{-1}$, which is close to 54.6(1)$\mu \text{S} \text {cm}^{-1} \text {min}^{-1}$, the corresponding rate of $\sigma ^{\mathrm{Eq.3}}_{\text{i}}$(t, H, Case).

Fourth, after the cessation of ${\text{CaCl}}_{2\mathrm{(aq)}}$ addition, both $\sigma ^{\mathrm{Eq.4}}_{\text{i}}$(H, Case) and $\text{pH} ^{\mathrm{Eq.4}}_{\text{i}}$(H, Case) tend towards steady-state values, though $\sigma ^{\mathrm{Eq.4}}_{\text{i}}$(H, Case) shows a slight linear increase while $\text{pH} ^{\mathrm{Eq.4}}_{\text{i}}$(H, Case) shows a slight decrease. The ultimate values in the “steady state” are dictated, mostly, by the chemistry of predominant NaCl. The weak differences in the steady-state parameters are attributed to challenges in ensuring identical initial conditions (e.g., solution concentration), the exact duration of ${\text{CaCl}}_{2\mathrm{(aq)}}$ addition, or stability of control parameters. For $\sigma ^{\mathrm{Eq.4}}_{\text{i}}$(H, Case) curves, these differences might be amplified by normalizing to $\sigma _{25 ^{\circ }{\text{C}}}$. A better analysis of these differences requires improvements to the current experimental setup.

Fifth, no correlation was observed between the (H, Case) pair and any of $\sigma ^{\text{React}}$(H, Case) in Fig. 3a and 4b, or $\text{pH} ^{\text{React}}$(H, Case) in Figs. 3b and 4c. This indicates that neither H nor Case has any discernible influence on ion strength, ion mobility, or [${\text{H}}^{+}$] and [$\text{OH}^{-}$]. For the weak differences, see the above discussion on the differences among the steady-state parameter values.

Finally, we observed no direct interplay between the magnetic and pH effects; however, the range and evolution of pH are observed to be reaction-dependent. Eq. (3) involves the dissociation of ${\text{HCO}_{3}^{-}}$ and the release of $\text{CO}_{2}$^63^, which contribute to a strong decrease in pH, as shown in Fig. 3b. This is not the case for Eq. (4), where the evolution of pH and the involved range differ, as seen by comparing Figs. 3b and 4c. As discussed in Section “Discussion”, such differences lead to marked variations in kinetics, stability, microstructure, and phase content.

**Fig. 5:**
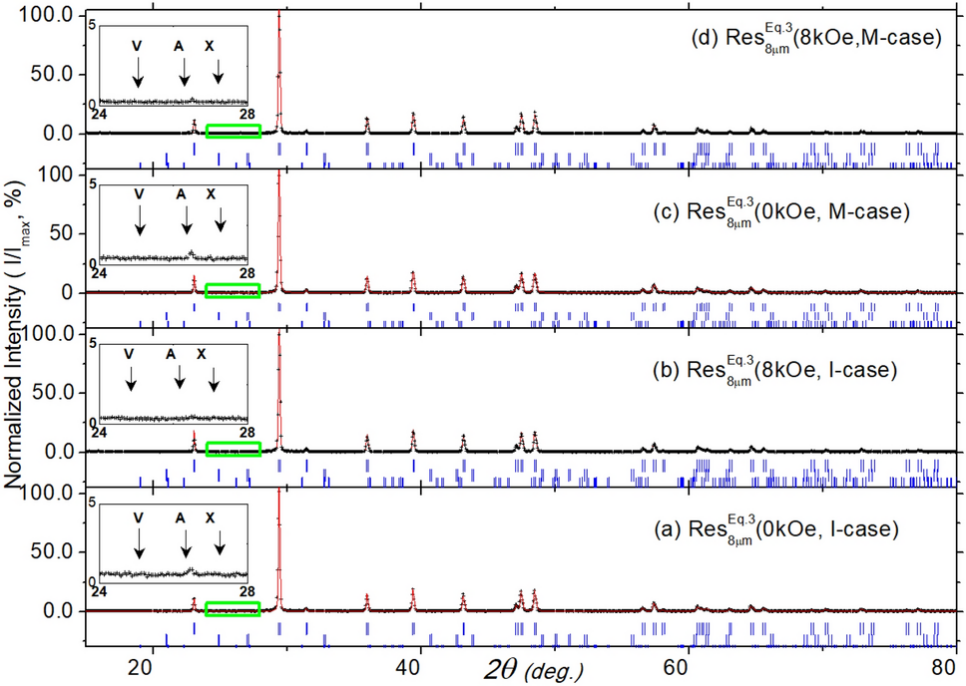
The analyzed diffractograms of ${\text {Res}}_{8\mu \text {m}}^{\text {Eq.3}}$(H, Case) with (**a**) H = 0.0 kOe and Case = I-case, (**b**) H = 8.0 kOe and Case = I-case, (**c**) H = 0.0 kOe and Case = M-case, and (**d**) H = 8.0 kOe and Case = M-case. All intensities are normalized by the maximum intensity of the majority calcite phase, $\text{I}_{\text{max}}$. Each *Inset* is an expansion of the region showing the expected Bragg peaks of the minority phases (vaterite $\big ($V,(100)$\big )$, aragonite $\big ($A,(111)$\big )$, and their overlap, $\big ($X: V(101), A(102)$\big )$. The weak Bragg peak at $26.5^\circ$ corresponds to an unidentified spurious phase.

**Fig. 6:**
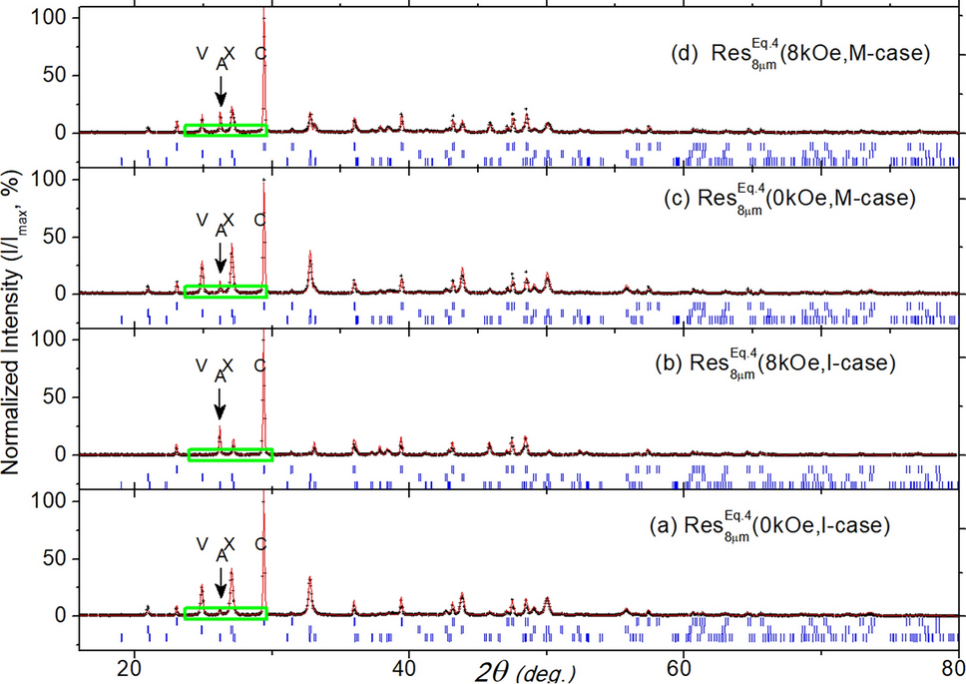
The refined diffractograms of ${\text {Res}}_{8\mu \text {m}}^{\text {Eq.4}}$(H, Case) with (**a**) H = 0.0 kOe and Case = I-case, (**b**) H = 8.0 kOe and Case = I-case, (**c**) H = 0.0 kOe and Case = M-case, and (**d**) H = 8.0 kOe and Case = M-case. All intensities are normalized by the maximum intensity of the majority calcite phase, $\text{I}_{\text{max}}$. The *green rectangles* highlight the Bragg peaks of calcite $\big ($C, (104)$\big )$, vaterite $\big ($V,(100)$\big )$, aragonite $\big ($A,(111)$\big )$, or the overlap of aragonite and vaterite (X, (101),(102)$\big )$.

### Crystalline structural properties analysis

#### Structural properties of ${\text {Res}}_{\text {Filt/evap}}^{\text {Eq.3}}$

The crystalline structures of representative ${\text {Res}}_{8\mu \text {m}}^{\text {Eq.3}}$(H, Case), shown in Fig. 5 and Table 1, are single-phase calcite, with no observable peaks related to vaterite or aragonite (see insets of Fig. 5). The refined cell parameters, averaged over all ${\text {Res}}_{8\mu \text {m}}^{\text {Eq.3}}$(H, Case) samples, are $\bar{\text{a}} \,=\, 4.990(1)$ Å and $\bar{{\text{C}}} \,=\, 17.072(2)$ Å, which align well with reported values^64^. Using the Scherrer equation, the average diameter of the crystalline domain is $\bar{\text{D}} = 544(90)$ Å.; it is recalled that this estimation is affected by both the process of crystallization and the method of sample preparation for X-ray diffraction measurement.

The refined diffractograms of ${\text {Res}}_{0.22\mu \text {m}}^{\text {Eq.3}}$(H, Case) samples (as shown in Fig.SM1) also indicate a single-phase calcite, with average parameters $\bar{\text{a}} = 4.990(2)$ Å, $\bar{{\text{C}}} = 17.070(9)$ Å, and $\bar{\text{D}} = 246(11)$ Å. On the other hand, the refined diffractograms of ${\text {Res}}_{\text {evap}}^{\text {Eq.3}}$(H, Case) (as shown in Fig. SM3 and Table 1) reveal a multi-phase structure. The major phase is halite [NaCl, $\bar{\text{a}} = 5.644(1)$ Å, $\bar{\text{D}} = 500(8)$ Å, $\bar{{\text{C}}} = 86(2)\%$], with minor phases of calcite [$\bar{{\text{C}}} = 2(1)\%$], vaterite [$\bar{{\text{C}}} = 3(2)\%$], and aragonite [$\bar{{\text{C}}} = 9(1)\%$], all having cell parameters consistent with reported values^64–66^. The presence of non-calcite polymorphs is attributed to the rise in temperature above $50^\circ$C during the evaporation process^52,67^.

Finally, no noticeable correlation is observed between each (H, Case) pair and the structural properties of ${\text {Res}}_{\text {Filt/evap}}$(H, Case) (see Section “Discussion”).

#### Structural properties of ${\text {Res}}_{\text {Filt/evap}}^{\text {Eq.4}}$

On analyzing and comparing the structural properties of ${\text {Res}}_{\text {Filt/evap}}^{\text {Eq.4}}$ with those of ${\text {Res}}_{\text {Filt/evap}}^{\text {Eq.3}}$, one observes that:

First, most of the structural properties of ${\text {Res}}_{8\mu \text {m}}^{\text {Eq.4}}$, shown in both Fig. 6 and Table 1, manifest a triphasic structure, in sharp contrast with the single calcite phase observed for ${\text {Res}}_{8\mu \text {m}}^{\text {Eq.3}}$. These phases are calcite $\big (\bar{\text{a}} = 4.992(3)$ Å, $\bar{{\text{c}}} = 17.071(8)$ Å, $\bar{\text{D}} = 554(74)$ Å, $\bar{{\text{C}}} = 44(8)\%\big )$, vaterite $\big (\bar{\text{a}} = 4.129(2)$ Å, $\bar{{\text{c}}} = 8.477(5)$ Å, $\bar{\text{D}} = 684(40)$ Å, $\bar{{\text{C}}} = 37(4)\%\big )$, and aragonite $\big (\bar{\text{a}} = 5.753(3)$ Å, $\bar{{\text{b}}} = 4.954(3)$ Å, $\bar{{\text{c}}} = 7.974(8)$ Å, $\bar{\text{D}} = 900$ Å, $\bar{{\text{C}}} = 19(3)\%\big )$. The presence of these phases is related to the high pH value, which favors the formation of vaterite and aragonite^52,60^. The stabilization of non-calcite phases, instead of forming only the most stable calcite, may be due to kinetic or chemical stabilization (as seen in aragonite shells)^60^.

Second, there is a strong correlation between the polymorphic phase content and field strength (see Table 1). Zero-field diffractograms are dominated by vaterite and calcite phases, with aragonite as a minority phase. In contrast, the diffractograms of field-treated samples show a significant reduction in vaterite and an enhancement in aragonite. This trend has been previously reported^4,5,68^. We argue that an applied field causes agglomeration of magnetic particulates, which act as templates favoring the formation of aragonite.

Third, the structures of ${\text {Res}}_{0.22\mu \text {m}}^{\text {Eq.4}}$(H, Case), shown in Fig.SM2 and Table 1, display the same above-mentioned field dependence. In the zero-field state, vaterite $\big (\bar{\text{a}} = 4.128(1)$ Å, $\bar{{\text{c}}} = 8.476(3)$ Å, $\bar{\text{D}} = 563(196)$ Å$\big )$ and calcite $\big (\bar{\text{a}} = 4.991(2)$ Å, $\bar{{\text{c}}} = 17.065(6)$ Å, $\bar{\text{D}} = 509(81)$ Å$\big )$ are dominant, while aragonite $\big (\bar{\text{a}} = 5.752(3)$ Å, $\bar{{\text{b}}} = 4.962(2)$ Å, $\bar{{\text{c}}} = 7.971(7)$ Å, $\bar{\text{D}} = 1080(100)$ Å$\big )$ is a minority phase. When a field of 8 kOe is applied, vaterite decreases $\big (\bar{{\text{C}}} = 51(6)\% \rightarrow 17(14)\%\big )$ while aragonite $\big (\bar{{\text{C}}} = 10(2)\% \rightarrow 31(9)\%\big )$ and calcite $\big (\bar{{\text{C}}} = 39(4)\% \rightarrow 52(6)\%\big )$ increase.

Finally, the refined diffractograms of ${\text {Res}}_{\text {evap}}^{\text {Eq.4}}$(H, Case), shown in Fig. SM4 and Table 1, indicate a polyphase structure. Besides the majority halite phase $\big (\bar{\text{a}}$ = 5.649(7) Å, $\bar{\text{D}}$ =  466(129) Å$\big )$, there are minority phases of calcite, vaterite, and aragonite, each with cell parameters matching reported values^64–66^. Interestingly, both the insets of Fig. SM4 and Table 1 show a much lower presence of aragonite compared to ${\text {Res}}_{\text {evap}}^{\text {Eq.3}}$(H, Case), even though both residues underwent the same evaporation procedures. Additionally, there is no clear field-dependent trend in the intensities of vaterite and aragonite, in contrast to the cases of ${\text {Res}}_{\text {Filt}}^{\text {Eq.4}}$(H, Case).

**Fig. 7:**
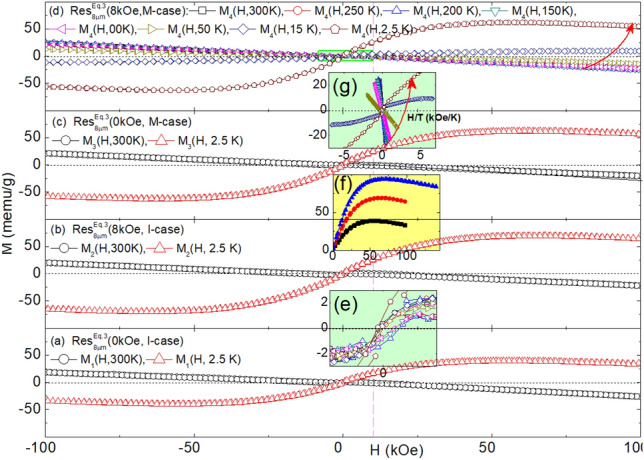
Magnetization Isotherms of ${\text {Res}}_{8\mu \text {m}}^{\text {Eq.3}}$(H, Case): (**a**) ${\text{M}}_1$(H,T = 300, 2.5   K) of ${\text {Res}}_{8\mu \text {m}}^{\text {Eq.3}}$(0kOe, I-case). (**b**) ${\text{M}}_2$(H,T = 300, 2.5 K) of ${\text {Res}}_{8\mu \text {m}}^{\text {Eq.3}}$(8kOe, I-case). (**c**) ${\text{M}}_3$(H,T = 300, 2.5 K) of ${\text {Res}}_{8\mu \text {m}}^{\text {Eq.3}}$(0kOe, M-case). (**d**) ${\text{M}}_4$(H,T) of ${\text {Res}}_{8\mu \text {m}}^{\text {Eq.3}}$(8kOe, M-case) wherein T = 300, 250, 200, 150, 100, 50, 15, 2.5K. The red curved arrow illustrates the trend in the evolution of magnetization isotherms as the temperature decreases. (**e**) Expansion of ${\text{M}}_4$(H,T) of ${\text {Res}}_{8\mu \text {m}}^{\text {Eq.3}}$(8kOe, M-case) of *panel d* indicating a weak magnetic hysteresis. As evident, there are differences in the remnant magnetization, ${\text{M}}_{\text{rem}}$, and coercive field, ${\text{H}}_{\text{coer}}$. (**f**) First quadrant of ${\text{M}}_\text{1}$(${\text{H}} \le$100  kOe,T = 2.5   K,$\blacksquare$) of *panel (a)*, ${\text{M}}_2$(${\text{H}} \le$100   kOe,T = 2.5   K, ) of *panel (b)*, and ${\text{M}}_2^{\mathrm{2-run}}$(${\text{H}} \le$140kOe,T = 2.5   K, ), manifesting the linear decrease in magnetization as field increases above saturation. (**g**) The same ${\text{M}}_4$(H,T) curves of *panel d* but plotted against ${{\text{H}}}/{{\text{T}}}$ wherein T = 300, 250, 200, 150, 100, 50, 15, 2.5K.

**Fig. 8:**
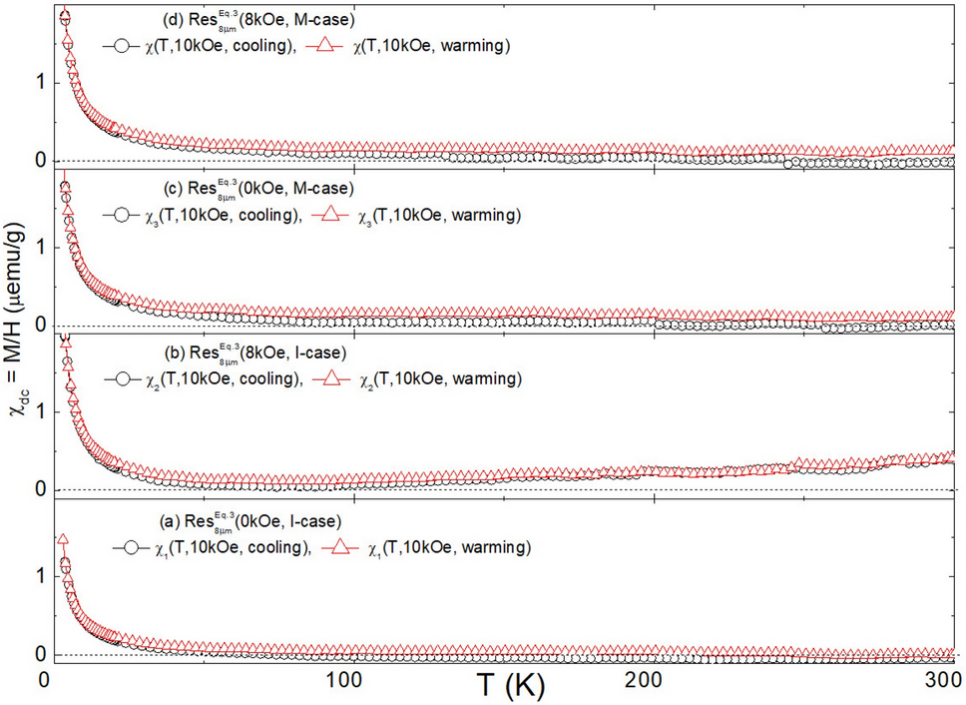
DC susceptibilities of ${\text {Res}}_{8\mu \text {m}}^{\text {Eq.3}}$(H, Case): (**a**) $\chi _{1}$(T, 10kOe, cooling/warming) of ${\text {Res}}_{8\mu \text {m}}^{\text {Eq.3}}$(0kOe, I-case). (**b**) $\chi _{2}$(T, 10kOe, cooling/warming) of ${\text {Res}}_{8\mu \text {m}}^{\text {Eq.3}}$(8kOe, I-case). (**c**) $\chi _{3}$(T, 10kOe, cooling/warming) of ${\text {Res}}_{8\mu \text {m}}^\text {{Eq.3}}$(0kOe, M-case). (**d**) $\chi _{4}$(T, 10kOe, cooling/warming) of ${\text {Res}}_{8\mu \text {m}}^{\text {Eq.3}}$(8kOe, M-case).

**Fig. 9:**
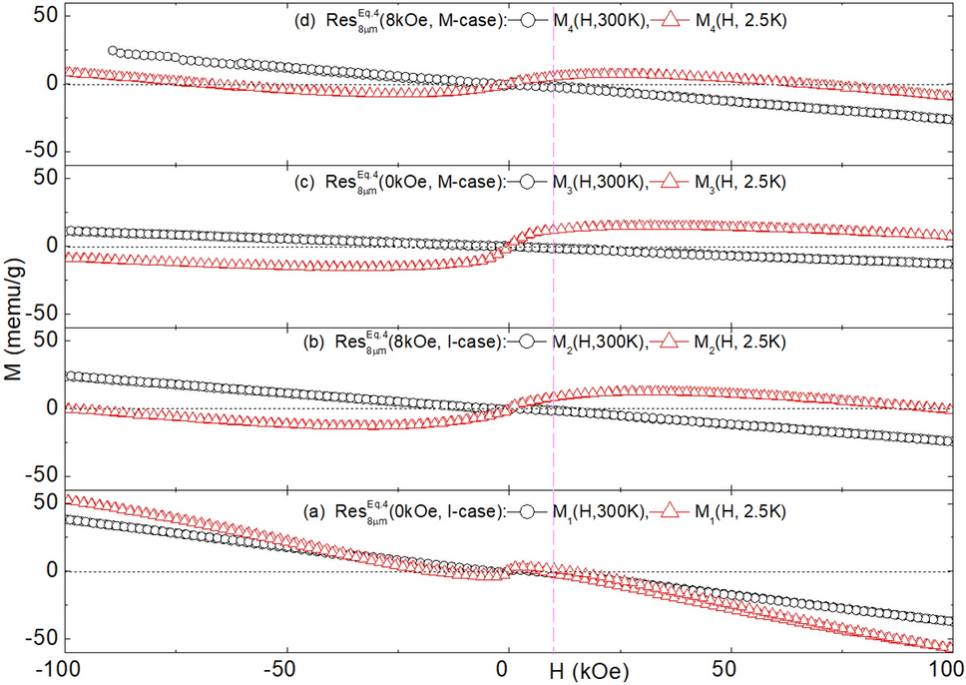
Magnetizations of ${\text {Res}}_{8\mu \text {m}}^{\text {Eq.4}}$(H, Case): (**a**) ${\text{M}}_1$(H,T = 300, 2.5 K) of ${\text {Res}}_{8\mu \text {m}}^{\text {Eq.4}}$(0kOe, I-case). (**b**) ${\text{M}}_2$(H,T = 300, 2.5 K) of ${\text {Res}}_{8\mu \text {m}}^{\text {Eq.4}}$(8kOe, I-case). (**c**) ${\text{M}}_3$(H,T = 300, 2.5 K) of ${\text {Res}}_{8\mu \text {m}}^{\text {Eq.4}}$(0kOe, M-case). (**d**) ${\text{M}}_4$ (H,T = 300, 2.5 K) of ${\text {Res}}_{8\mu \text {m}}^{\text {Eq.4}}$(8kOe, M-case).

**Fig. 10:**
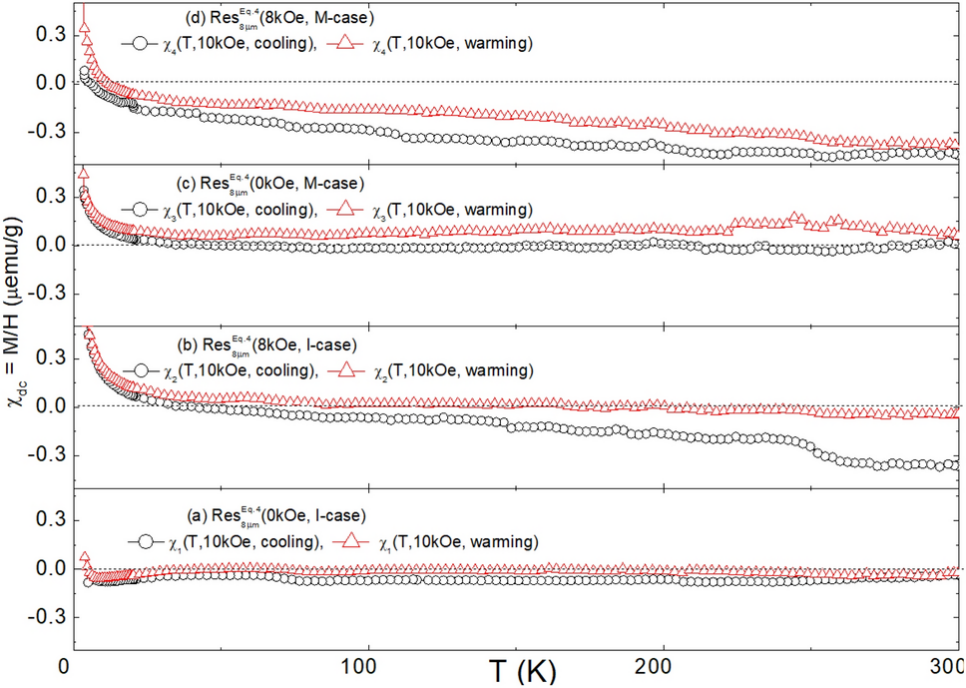
DC susceptibilities of ${\text {Res}}_{8\mu \text {m}}^{\text {Eq.4}}$(H, Case): (**a**) $\chi _{1}$(T, 10kOe, cooling/warming) of ${\text {Res}}_{8\mu \text {m}}^{\text {Eq.4}}$(0kOe, I-case). (**b**) $\chi _{2}$(T, 10kOe, cooling/warming) of ${\text {Res}}_{8\mu \text {m}}^{\text {Eq.4}}$(8kOe, I-case). (**c**) $\chi _{3}$(T, 10kOe, cooling/warming) of ${\text {Res}}_{8\mu \text {m}}^{\text {Eq.4}}$(0kOe, M-case). (**d**) $\chi _{4}$(T, 10kOe, cooling/warming) of ${\text {Res}}_{8\mu \text {m}}^{\text {Eq.4}}$(8kOe, M-case).

### Magnetic properties analysis

#### Magnetization of residues obtained from $\text{NaHCO}_{3}$ route (Eq. 3): $\text {M}\big (\text {H, T, Res}_{\text {Filt}}^{\text {Eq.3}}\text {(H, Case)}\big )$

Evolution of representative magnetization isotherms, $\text {M}\big (\text {H, T, Res}_{\text {Filt}}^{\text {Eq.3}}\text {(H, Case)}\big )$, is shown in Fig. 7 (see also Fig. SM5). For each (H, Case), the room-temperature contribution is dominantly diamagnetic, superimposed on a weak ferromagnetic contribution, such as the one shown in Fig. 7e. The latter exhibits hysteresis with weak ${\text{m}}_{\text{rem}}$ and ${\text{H}}_{\text{coer}}$.

On lowering the temperature to 2.5 K, each $\text {M}\big (\text {H, 2.5\,K, Res}_{\text {Filt}}^{\text {Eq.3}}\text {(H, Case)}\big )$ reveals a strong ferromagnetic contribution with saturation occurring only at very high fields. Above saturation, as shown in Fig. 7f, the diamagnetic contribution emerges as a high-field *linear-in-H* component with a negative slope approximating that of the room-temperature diamagnetic contribution.

The thermal evolution of $\text {M}\big (\text {H, 2.5} \le \text {T} \le \text {300\,K, Res}_{\text {Filt}}^{\text {Eq.3}}(\text {H, Case})\big )$ is well depicted in Fig. 7(d). The predominantly diamagnetic and quasi *linear-in-H* high-temperature behavior remains almost constant down to 50K. Below this temperature, the ferromagnetic contribution is enhanced, more sharply below 15 K. These features are also evident in $\chi \big (\text {10kOe, T, Res}_{\text {Filt}}^{\text {Eq.3}}\text {(H, Case)}\big )$ in Figs. 8 and SM6. Interestingly, the plot of $\text {M}\big (\text {H, 2.5} \le \text {T} \le \text {300K, Res}_{\text {Filt}}^{\text {Eq.3}}\text {(H, Case)}\big )$ against ${{\text{H}}}/{{\text{T}}}$ in Fig. 7(g) reveals that the rapid low-temperature rise in susceptibility and magnetization is not solely due to paramagnetism. If it were, the Langevin (or Brillouin for quantum paramagnetism) function would collapse all these curves onto each other^69,70^. Additionally, there is no sign of a characteristic spin-flop, a hallmark of magnetization curves within the ordered range of an antiferromagnet. Therefore, it is inferred that the magnetic signals are a sum of diamagnetic, paramagnetic, and ferromagnetic-like contributions.

The diamagnetic contribution, whenever dominant, evolves linearly with the magnetic field. Its average susceptibility, $\chi _{\text{dc}} = \frac{\partial {\text{M}}}{\partial {\text{H}}} \big | _{\text{300K}}$, is found to be $-3.2(6) \times 10^{-7}$ emu/g, as seen in Figs. 7a, which agrees with the average susceptibility of calcite $\chi = -3.6(1) \times 10^{-7}$ emu/g^71,72^. This confirms, as already indicated by XRD structural analysis, that the major phase is calcite. Calcite is known for its ability to incorporate transition metal ions, particularly Fe, into the ${\text{Ca}^{2+}}$ site, giving an additional paramagnetic contribution^71,72^.

The ferromagnetic-like contribution, indicative of nano-ferromagnetic particles with ${\text{T}}_{\text{C}} > 300$ K, gives rise to a relatively strong saturation, characteristic hysteresis features, as in Fig. 7e, and a noticeable zero-field cooling (ZFC) and field-cooling (FC) behavior. The progressive increase in saturation, decreasing remanence, and diminishing coercivity as temperature decreases suggest nanosized superparamagnets with distribution in their size and blocking temperature, ${\text{T}}_{\text{B}}$^69^. Furthermore, for ${\text{T}} < 50$ K, progressive blocking leads to enhanced magnetization and susceptibility. No signs of collective freezing, magnetic order, or high-field open hysteresis loops were observed, suggesting a wide separation and weak interactions among these superparamagnetic contaminants^69^.

Since none of the chemical components in Eq. (3) are intrinsically magnetic, the *positive-in-H* magnetic signals (especially the ferromagnetic-like ones) must be due to magnetic contamination (see Table SM1). Empirically, the ratio of the intensity of this contaminating signal to that of the diamagnetic signal varies considerably across Fig. 7 (see also Fig.SM5). This variability is attributed to the random concentration of magnetic contaminants (see Table SM1). Despite this randomness in content and size distribution across different residues, there is a strong overall similarity between the curves of $\text {M}\big (\text {H, 2.5} \le \text {T} \le \text {300K, Res}_{\text {Filt}}^{\text {Eq.3}}\text {(H, Case)}\big )$. Similarly, for those of $\chi \big (\text {10kOe, T, Res}_{\text {Filt}}^{\text {Eq.3}}\text {(H, Case)}\big )$.

#### Magnetization of residues obtained from ${\text{Na}_{2}\text{CO}_{3}}$ route (Eq. 4): $\text {M}\big (\text {H, T, Res}_{\text {Filt}}^{\text {Eq.4}}\text {(H, Case)}\big )$

The evolution of representative magnetization and susceptibility of ${\text {Res}}_{\text {Filt}}^{\text {Eq.4}}$(H, Case) is shown in Figs. 9–10 (see also Figs.SM7-SM8). The overall trend is very similar to that of the corresponding ${\text {Res}}_{\text {Filt}}^{\text {Eq.3}}$(H, Case), indicating that the source of the magnetic contamination is likely the same. Nevertheless, there are notable differences in the magnetic properties. For instance, the intensities of the magnetic signals in Figs. 9–10 and SM7-SM8, although varying from sample to sample, are on average less than half of those observed in ${\text {Res}}_{\text {Filt}}^{\text {Eq.3}}$(H, Case). Coincidentally, the phase content of calcite also drops by half (see Table 1). However, these two trends do not appear to be correlated.

To clarify these differences, we investigated the possibility that they originate from the reactants used in Eqs. (3)–(4). The magnetic properties of ${\text{NaHCO}_\text{3}}$, ${\text{Na}_{2}\text{CO}_{3}}$, and ${{\text{CaCl}}_{2}}$ (measured following the experimental procedures described in Section “Methods”) are weak and paramagnetic: $\chi _{\text{dc}}^{{\text{CaCl}}_\text{2}}\mathrm{(10kOe, 300K)} \approx +2.3(4) \times 10^{-7}$ emu/g (the reported value is $-4.9 \times 10^{-7}$ emu/g)^73^. $\chi _{\text{dc}}^{\text{Na}_{2}\text{CO}_\text{3}}\mathrm{(10kOe, 300K)} \approx +5 \times 10^{-13}$ emu/g (the reported value is $-3.9 \times 10^{-7}$ emu/g)^73^. $\chi _{\text{dc}}^{\text{NaHCO}_\text{3}}\mathrm{(10kOe, 300K)} \approx +6(2) \times 10^{-8}$ emu/g.

This paramagnetism is likely due to the presence of contaminating paramagnetic ions, most probably transition metals, incorporated within the cationic sites of the reactants. Even if these paramagnetic ions are incorporated into the products-particularly ${\text{CaCO}}_{3}$-of Eq. (4), they remain paramagnetic and contribute to the weak paramagnetism of the final products. Therefore, we associate the ferromagnetic-like contributions observed in all residues to contaminating magnetic particulates, most likely formed within the experimental setup depicted in Fig. 2 (see Section “On the dependence of non-uniform field and contaminant content”).

No influence of H or Case variation was observed on $\text {M}\big (\text {H, T, Res}_{\text {Filt}}^{\text {React}}\text {(H, Case)}\big )$ or $\chi \big (\text {H, T, Res}_{\text {Filt}}^{\text {React}}\text {(H, Case)}\big )$ (see Section“ Discussion”). As discussed above, the ferromagnetic-like contribution is governed by random concentrations of magnetic contaminants. Similarly, the intensity and slope of the diamagnetic contribution, while varying slightly across ${\text {Res}}_{\text {Filt}}^{\text {React}}$(H, Case), show no correlation with the (H, Case) pair.

**Fig. 11:**
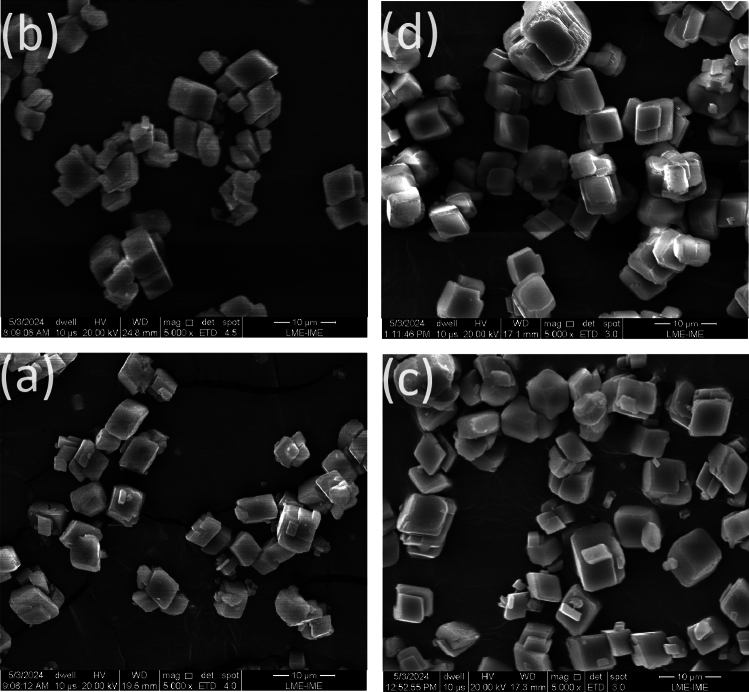
Morphologies and polymorphism of ${\text {Res}}_{\text {8}\mu \text {m}}^{\text {Eq.3}}$(H, Case) when varying H or Case: (**a**) SEM micrographs of ${\text {Res}}_{8\mu \text {m}}^{\text {Eq.3}}$(0kOe, I-case). (**b**) SEM micrographs of ${\text {Res}}_{8\mu \text {m}}^{\text {Eq.3}}$(8kOe, I-case). (**c**) SEM micrographs of ${\text {Res}}_{8\mu \text {m}}^{\text {Eq.3}}$(0kOe, M-case). (**d**) SEM micrographs of ${\text {Res}}_{8\mu \text {m}}^{\text {Eq.3}}$(8kOe, M-case).

**Fig. 12:**
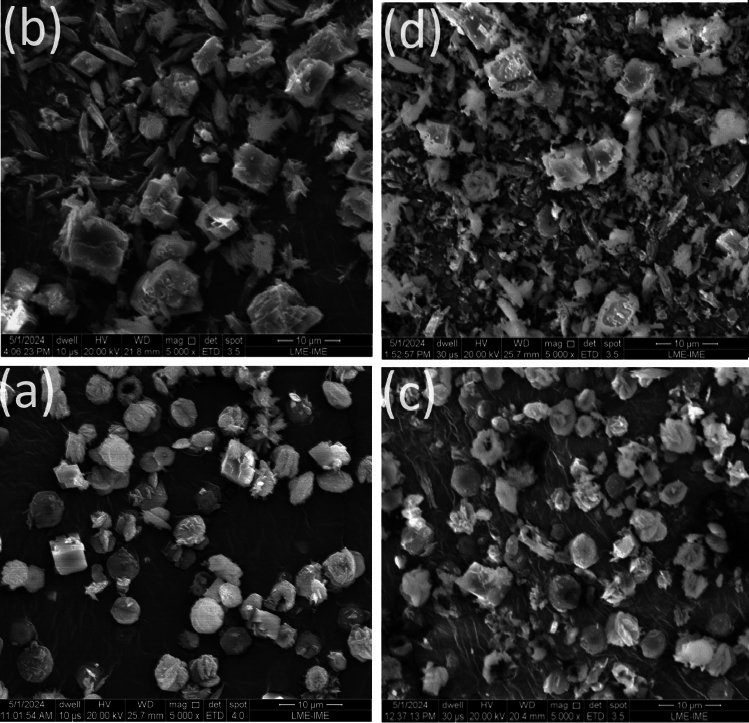
Morphologies and polymorphism of ${\text {Res}}_{8\mu \text {m}}^{\text {Eq.4}}$(H, Case) when varying H or Case: (**a**) SEM micrographs of ${\text {Res}}_{8\mu \text {m}}^{\text {Eq.4}}$(0kOe, I-case). (**b**) SEM micrographs of ${\text {Res}}_{8\mu \text {m}}^{\text {Eq.4}}$(8kOe, I-case). (**c**) SEM micrographs of ${\text {Res}}_{8\mu \text {m}}^{\text {Eq.4}}$(0kOe, M-case). (**d**) SEM micrographs of ${\text {Res}}_{8\mu \text {m}}^{\text {Eq.4}}$(8kOe, M-case).

### Morphology, polymorphism, and elemental analysis

As mentioned above, microstructural analysis of n the product ${\text{CaCO}}_{3}$ is particularly suitable for studying the magnetic field’s impact on scale formation. Below, we analyze the microstructure and elemental composition of ${\text {Res}}_{\text {Filt}}^{\text {React}}$(H, Case, Contaminant) as obtained from a SEM technique equipped with an EDS option. Representative results are shown in Figs. 11, 12, SM9–SM10, SM18, and Table SM1. The SEM micrographs of ${\text {Res}}_{\text {Filt}}^{\text {Eq.3}}$(H, Case), shown in Figs. 11, SM9, indicate a monophase with a cuboid morphology and a stoichiometry, as detailed in Table SM1, approximating the theoretically expected values. There is no significant change in polymorphic content or morphology when varying H or cell casing.

In contrast, Figs. 12 and SM10 display SEM micrographs of ${\text {Res}}_{\text {Filt}}^{\text {Eq.4}}$(H, Case), which show distinctly different structures compared to ${\text {Res}}_{\text {Filt}}^{\text {Eq.3}}$(H, Case). In addition to cuboid calcite, formations of vaterite (e.g., spherulites, star-like, flower-like, or cauliflower-like) and aragonite (e.g., elongated needle-like or bundles) are observed. Notably, the microstructure of ${\text {Res}}_{\text {Filt}}^{\text {Eq.4}}$(H, Case) is significantly influenced by variations in pH, contamination, or magnetic field, but not by the cell casing, as can be seen when comparing Fig. 12a,b with Fig. 12c,d.

Figure 12a,c shows that the micrographs of ${\text {Res}}_{8\mu \text {m}}^{\text {Eq.4}}$(0kOe, Case) are dominated by spheroidal or star-like vaterite along with a minority of cuboidal calcite. In contrast, those of ${\text {Res}}_{8\mu \text {m}}^{\text {Eq.4}}$(8kOe, Case), depicted in Fig. 12b,d, reveal a significant reduction in vaterite and an enhancement in both aragonite and calcite. These findings align with the structural analysis discussed in Section “Crystalline structural properties analysis” and Table 1.

The EDS analysis of ${\text {Res}}_{\text {Filt}}^{\text {React}}$(H, Case), with representative results shown in Fig.SM18 and Table SM1, indicates the presence of the major constituents and some contaminating trace elements. Compared to the ideal values listed in the last row of Table SM1, the mass percentages of the major constituents vary across the same sample and among different residues. The values provided are averages, with large standard deviations (given in parentheses), suggesting some degree of non-uniform stoichiometry within the samples. Similarly, the type and concentration of trace elements vary within the same sample and across different residues, with no perceptible correlation with reaction type, H, or Case.

Generally, the normalized mass percentage of trace elements in the studied samples is low; often, the content of a *3d* contaminant is below 1000 ppm, as shown in Table SM1. The magnetic *3d* contaminants likely contribute to the observed *positive-in-H* signal. Some of these elements may exist as paramagnetic substituents or interstitials within the ${\text{CaCO}}_{3}$ lattice, while others could be present as separate nano-sized superparamagnetic particles.

While EDS analysis of Fig.SM18 and Table SM1 confirms the presence of magnetic contamination, it does not directly correlate the content of magnetic *3d* trace elements to the overall magnetization of the sample. This is probably because some of these magnetic trace elements, which contribute to the heterogeneous nucleation, may reside beyond the reach of the EDS probe. In our case, the EDS penetration depth is estimated to be less than $1\,\mu \text {m}$, potentially missing deeper-seated contaminants.

### On the dependence of non-uniform field and contaminant content

Equations (1 and 2) highlight the distinct influences of uniform and non-uniform magnetic fields. Moreover, Fig. 2a,b,d indicate that the spatial range where the field is uniform comprises the cylinder bounded by the circular surfaces of the opposing electromagnet’s poles. Directly outside these surfaces, where the cylinder intersects the electrolytic cell, there is a strong $\nabla {\text{B}}$. Thus, for all the mentioned experiments, the moving *supersaturated-contaminant-bearing solution* is exposed to both uniform and non-uniform fields. Our current setup does not permit the establishment of a strictly uniform magnetic field but does allow the addition of an extra non-uniform field, as shown in Fig. 2c. Using these field arrangements and following the procedures in Section “Methods”, we analyzed the impact of adding such an extra non-uniform field.

During the mounting of the additional non-uniform field arrangement, we replaced the pressure sensor (manometer) due to partial corrosion. Consequently, the following experiments were conducted under a combination of an additional non-uniform field ($\nabla {\text{B}}$) and reduced contamination (less contaminant). The results obtained under these conditions are summarized below:

First, the evolution of $\sigma ^{\text{React}}$(H, Case,less-contaminant) and $\text{pH} ^{\text{React}}$(H, Case,less-contaminant), shown in Fig. SM11, are almost identical to the corresponding evolutions in Figs. 3, 4. This confirms the previously noted absence of a field influence on ion concentration and mobility within our experimental conditions.

Second, the structural analysis of ${\text {Res}}_{\text {Filt}}^{\text {Eq.3}}$(H, Case,less-contaminant), shown in Fig.SM12 and Table 1, indicates a single-phase calcite, consistent with those in Fig. 5. Similarly, the micrographs analysis of ${\text {Res}}_{\text {Filt}}^{\text {Eq.3}}$(H, Case, less-contaminant), shown in Fig.SM16 and Table SM1, reveal a single-phase calcite character, just as in Fig. 11. But, Table SM1 indicates that the trace elements in ${\text {Res}}_{\text {Filt}}^{\text {Eq.3}}$(H, Case, less-contaminant) are slightly lower than those in ${\text {Res}}_{\text {Filt}}^{\text {Eq.3}}$(H, Case). These observations confirm the absence of a field/contamination-induced microstructure-shaping effect for all ${\text {Res}}_{\text {Filt}}^{\text {Eq.3}}$(H, I-case), regardless of field, Case, or contamination variations.

Third, the crystalline structure of ${\text {Res}}_{8\mu \text {m}}^{\text {Eq.4}}$(H, Case,less-contaminant), shown in Fig.SM13 and Table 1, consists of both calcite $\big (\bar{\text{a}}$  =  4.993 Å, $\bar{{\text{c}}}$  =  17.073 Å, $\bar{{\text{C}}} = 32\%\big )$ and vaterite $\big (\bar{\text{a}}$  =  4.130 Å, $\bar{{\text{c}}}$  =  8.478 Å, $\bar{{\text{C}}} \,=\, 68\%\big )$, but almost no aragonite. Similarly, the diffractograms of ${\text {Res}}_{0.22\mu \text {m}}^{\text {Eq.4}}$(H, I-case), shown in Fig.SM13, reveal the same biphasic structure: calcite $\big (\bar{\text{a}}$  =  4.993 Å, $\bar{{\text{c}}}$  =  17.073 Å, $\bar{{\text{C}}} \,=\, 17\%\big )$ and vaterite $\big (\bar{\text{a}}$  =  4.130 Å, $\bar{{\text{c}}}$  =  8.478 Å, $\bar{{\text{C}}}$  =  83%$\big )$. This biphasic character is also evident in the micrographs of Fig.SM17. Comparing this biphasic character with the triphasic character analyzed in § 2.2.2, and noting the reduced contamination shown in Table SM1, the triphasic-into-biphasic modification of ${\text {Res}}_{\text {Filt}}^{\text {Eq.4}}$(H, I-case) must be related to the reduction in magnetic contamination (see discussion in Section “Discussion”). Based on Table SM1, we were unable to conclusively affirm the presence and trend of a field/contamination-induced microstructure-shaping effect in ${\text {Res}}_{\text {Filt}}^{\text {Eq.4}}$(H, Case, less-contaminant). While the majority vaterite phase slightly decreases with H for Filt = $0.22\mu {\text{m}}$, it slightly increases for Filt = $8\mu {\text{m}}$. Further analysis is underway.

Fourth, the overall magnetic properties of ${\text {Res}}_{8\mu {\text {m}}}^{\text {Eq.3}}$(H, Case, less-contaminant), shown in Fig.SM14, are very similar to those in Figs. 7a,b and 8a,b. Similar observations apply to the overall magnetic properties of ${\text {Res}}_{0.22\mu {\text {m}}}^{\text {Eq.3}}$(H, Case, less-contaminant), comparing Fig.SM14(c,d) with Figs.SM5(a,b) and SM6(a,b). Noteworthy is the remarkable difference: the ratio of the positive-in-H contribution to the diamagnetic contribution-evident in the high-field ${\text{M}}$(H, T, 2.5K) and low-temperature $\chi$(T, 10kOe, cooling/warming) curves-is smaller in ${\text {Res}}_{\text {Filt}}^{\text {Eq.3}}$(H, Case, less-contaminant) due to the above-mentioned lower magnetic contamination (see Table SM1). This reduction of the positive contribution is particularly evident in the magnetic properties of ${\text {Res}}_{\text {Filt}}^{\text {Eq.4}}$(H, Case, contaminant): The ratio obtained from Fig.SM15 is much smaller than the ratios obtained from Figs. 9–10 and SM7.

Finally, Figs. SM14 and SM15 compare the magnetization of ${\text {Res}}_{\text {Filter}}^{\text {React}}(0, \text {I-case}, \text {contaminant})$ with that of ${\text {Res}}_{\text {Filter}}^{\text {React}}({\text{H}} + \nabla {\text{H}}, \text {I-case}, \text {contaminant})$. The introduction of ${\text{H}} + \nabla {\text{B}}$ shows no significant change in magnetization nor in the microstructural properties. We argue that a reduction or absence of magnetic contamination would lead to fewer or no field-responsive magnetic templates for the nucleation and growth of aragonite. Consequently, this would result in a lack of an indirect microstructure-shaping effect, as evident in Figs.SM13,SM17.

## Discussion

It is evident from the above that the necessary conditions for manifesting a field/contamination-induced microstructure-shaping effect are a higher pH environment and the availability of sufficient magnetic impurities that act as templates for heterogeneous nucleation and growth. The first condition is derived from the fact that such an effect is absent in all residues associated with Eq. (3) (pH < 8.5), regardless of magnetic contaminants. The second condition comes from the analysis of the magnetic properties of ${\text {Res}}_{\text {Filt}}^{\text {Eq.4}}$(H, Case, pH, Contaminant). For a sample with a triphasic structure and such a structure-shaping effect, the magnetization, as seen in Figs. 9, 10, shows a strong ferromagnetic-like contribution. In contrast, for a sample with a biphasic structure and no such effect, the magnetization, as seen in Fig.SM15, exhibits a much-reduced ferromagnetic-like contribution. These arguments suggest that controlling the indirect microstructure-shaping effect is possible only when the conditions concerning pH and magnetic contamination are met. Under these conditions, field control is indirectly achieved by manipulating the catalytic role of the magnetic impurities.

One prominent manifestation of the induced microstructure-shaping effect is the field control of the fractional content of aragonite. This effect is related to the sensitivity of the kinetics of aragonite formation to impurities^52^. The presence of magnetic contaminants, which can agglomerate under the influence of a field and act as templates, appears to trigger a partial destabilization of vaterite and favor the assisted stabilization of aragonite (see, e.g., Figs. 6 and 12). It is worth investigating whether the entire vaterite phase would eventually convert to aragonite over a prolonged period.

Interestingly, the onset of such an indirect microstructure-shaping effect is not reflected in the evolution of in-situ conductivity and pH monitoring curves, as the corresponding techniques only monitor ions content and transport properties. In contrast, this induced microstructure-shaping effect is evident in the evolution of ex-situ microstructural analysis, highlighting that the magnetic field’s influence is confined to the second subsystem, which contains the magnetic contaminants. This inference—that the impact of the magnetic field is on neutral particulates—is confirmed by the study of Torraca^74^, who reported on the evolution of the reaction in Eq. (3) through in-situ monitoring of the concentration and average diameter of precipitated ${\text{CaCO}}_{3}$ particles using an Optical Particle Size Analyzer. These experiments were carried out under both zero and applied magnetic fields. The two main findings are: (i) the reaction and precipitation of ${\text{CaCO}}_{3}$ occur during the period of ${{\text{CaCl}}_{2}}$ addition (e.g., the light blue area in Fig. 3); and (ii) an applied magnetic field leads to an earlier initiation of the nucleation process compared to the zero-field case. Both findings support our interpretation that magnetic fields induce agglomeration of magnetic contaminants, which act as additional heterogeneous nucleation centers and shorten the induction time.

Next, we discuss the influence of the cell casing on the evolution of the studied properties of a moving, uniform, supersaturated, contaminants-bearing solution. In this regard, we verified that (i) the applied magnetic field fully penetrates the stainless tube; (ii) the weight of the stainless tube before and after each experiment remains unchanged; and (iii) an inspection of the tube after the experiment showed no apparent corrosion or deposition on its inner surface. Based on these observations, we infer that the two field-induced forces described in Eqs. (1) and (2) are the same regardless of whether the casing is stainless-steel or insulating. However, as illustrated in Fig. 1d, the presence of a metallic casing leads to the short-circuiting of the induced electromotive force (EMF), which can, in specific cases, cause Faradaic reactions at the opposite boundaries of the cell^5,9^. This can result in deposition on one side^12,75^, corrosion on the other side, or the formation of small incipient particulates within the fluid that can act as templates or inhibitors^52^. In our setup, we do not expect any corrosion, release of nucleation centers from the tube into the fluid, or formation of protrusions on the inner surfaces of the SS-based cell housing. Furthermore, as discussed in the Introduction, the induced EMF, though giving rise to Hall voltage, has no practical impact on any stage of the scaling process. We extend both these arguments and our empirical observations to assert that the insertion of the stainless steel tube does not alter the evolution of conductivities, pH levels, magnetizations, or microstructures. This would certainly not be the case if the metallic tube could sustain a Faradaic reaction that allows, for example, the release of magnetic particulates into the bulk of the working fluid.

Finally, to clarify the ASMT controversy, we argue that the seemingly divergent standpoints arise because each perspective evaluates a specific working fluid (which is composed of a particular combination of the two above-mentioned idealized subsystems). At one extreme is the case of a non-contaminated supersaturated solution. Here, the magnetic field’s only influence arises from the Lorentz force, which, under typical laboratory conditions, does not significantly impact the scaling process. All experiments conducted with a working fluid approximating this limit support the standpoint that the field has no influence: ASMT is ineffective and unreliable. At the other extreme is the case of a mixture of a supersaturated solution and a suspension with a high concentration of magnetic contaminants. In this scenario, the magnetic contaminants, being manipulable by an externally applied magnetic force gradient, can act as templates. This allows the magnetic field to indirectly influence the heterogeneous nucleation and growth processes. All experiments conducted with such a working fluid support the standpoint that the magnetic field can influence the kinetics, stability, or microstructure of the scaling process: ASMT is effective and reliable.

## Summary and conclusions

To clarify the controversy surrounding the influence of an applied magnetic field on an inorganic scaling process, this study focused on ${\text{CaCO}}_{3}$ as a model scalant. Our findings highlight the combined roles of pH level, impurity type, and impurity content in determining the response of the different stages of scaling to an applied field. These findings are summarized below.

First, we employed both conductivity and pH to monitor how a field influences the properties of the ionic subsystem. We found no field or Case dependency in the evolution of $\sigma ^{\text{React}}$(H, Case) or $\text{pH}^{\text{React}}$(H, Case). This indicates that neither the ionic concentration (including [${\text{H}}^{+}$] and [$\text{OH}^{-}$]) nor ionic mobility depends on H or Case. Distinct evolution of $\sigma ^{\text{React}}$(H, Case) and $\text{pH}^{\text{React}}$(H, Case) were observed when varying React but hardly any when varying H or Case. Specifically, although both $\text{pH}^{\mathrm{Eq.3}}$(H, Case) and $\text{pH}^{\mathrm{Eq.4}}$(H, Case) decrease almost exponentially, the evolution of individual pH curves-their range, rate, and whether they follow a one-step or two-step process-differs significantly. Similarly, the evolution of $\sigma ^{\mathrm{Eq.3}}$(H, Case) differs significantly from that of $\sigma ^{\mathrm{Eq.4}}$(H, Case). For both techniques, monitoring solution of Eq. (3) suggests a single-step process, while monitoring Eq. (4) exhibits a two-step process. The differences in conductivity and pH evolution are accompanied by distinct differences in the evolution of nucleation, growth, and consequently, the microstructure of the resulting scale.

Second, the manifestation of a one-step process in Eq. (3) observed in in-situ monitoring techniques directly correlates with the single-phase calcite structure of ${\text {Res}}_{\text {Filt}}^{\text {Eq.3}}$ observed in ex-situ probing techniques. In contrast, the two-step process in Eq. (4), with the first event corresponding to vaterite/aragonite formation and the second event to calcite formation, correlates with the multi-phase structure of ${\text {Res}}_{\text {Filt}}^{\text {Eq.4}}$ observed in ex-situ probing techniques.

Third, these correlations emphasize the significant role of the pH environment. For a relatively low pH range, a single-step process and a monophase calcite microstructure are observed, which are insensitive to impurities or an applied magnetic field (no indirect microstructure-shaping effect). In contrast, for a higher pH level that favors the additional formation of vaterite or aragonite polymorphs, a two-step process and a polyphase microstructure are observed. Here, the significant catalytic role of contaminating templates in stabilizing the metastable vaterite and aragonite phases is emphasized.

Fourth, ${\text {Res}}_{\text {Filt}}^{\text {Eq.4}}$(H, Case, pH, Contaminant) exhibits a strong field/contamination-induced microstructure-shaping effect only when there are sufficient magnetic impurities. In such cases, the deposit ${\text {Res}}_{\text {Filt}}^{\text {Eq.4}}$(H, Case, pH, Contaminant) from the corresponding field-treated solution shows a triphasic structure, where vaterite is field-reduced, and aragonite is field-enhanced. In contrast, under conditions leading to a biphasic structure, no equally significant induced microstructure-shaping effect is observed.

Lastly, evaluating the field and thermal evolution of magnetization proved valuable in, e.g., it allowed us to infer that all magnetization curves can be decomposed into a sum of a diamagnetic signal originating from ${\text{CaCO}}_{3}$ polymorphs and a positive signal originating from magnetic contaminants. We suggest that a gradient-induced agglomeration of magnetic impurities affects the nucleation and growth processes, consequently influencing the corresponding microstructure. Combined with findings from conductivity, pH, diffraction, and SEM analyses, we conceptualize the working contaminants-bearing solution as an idealized homogeneous mixture of two subsystems: The first subsystem consists of a non-contaminated supersaturated solution containing diamagnetic multi-ionic components. The influence of a magnetic field is primarily expressed through the Lorentz force, which minimally impacts the microstructure of the scale, except in cases where extensive Faradaic reactions occur. The second subsystem consists of a suspension of spurious neutral entities, which can exhibit diamagnetic, paramagnetic, antiferromagnetic, or ferromagnetic-like properties. These entities serve as templates or inhibitors in the scaling process. Notably, the distribution/agglomeration of these entities, particularly the ferromagnetic-like particulates if present, can be significantly affected by an applied field. This allows for indirect manipulation of the microstructure of the scale: A field/contaminant-induced microstructure-shaping effect.

Our analysis indicates that the controversy can be clarified by recognizing that the viewpoint claiming ineffectiveness and unreliability refers to the situation where the magnetic field’s influence on a diamagnetic supersaturated solution is extremely weak. On the other hand, the viewpoint asserting effectiveness and reliability pertains to the magnetic field’s influence on a contaminated system. Since each side holds a valid aspect of the overall picture, the controversy is demystified.

Although we used ${\text{CaCO}}_{3}$ as a model for scaling, our arguments and conclusions extend to a wider range of scale formations. This generalization is particularly applicable to the regulatory effects of pH environments, the catalytic role of magnetic impurities, and the indirect influence of applied magnetic fields on the nucleation and growth of scales. Our findings provide insights that help explain and interpret much of the existing literature on these topics. Further extension and generalization of our analysis to other inorganic scales and similar scaling phenomena will be detailed in future publications.

## Methods

The field influence on the different stages of scaling within a *supersaturated-contaminants-bearing solution* can be investigated by monitoring the evolution of in-situ parameters (e.g., conductivity, pH, optical absorbance, particle number, or size distribution) and ex-situ parameters (e.g., elemental, magnetic, microstructural, or spectroscopic properties). During such experiments, it is common practice to control variable such as temperature (${\text{T}}$), hydrostatic pressure (${\text{P}}$), flow rate (${\text{F}}$), magnetic field, (${\text{H}}$) and cell casing (Case). In this study, we measured all these parameters using our custom-built, computer-interfaced experimental setup shown in Fig. 2a. In our experiments, all control parameters were held constant except the strength of the applied magnetic field (${\text{H}}\,=\,0.0$ or 8.0 kOe) and the type of cell casing: Case  =  Metallic (M-case) or Insulating (I-case).

The central component of the setup is the cell, which is positioned within the poles of an electromagnet as illustrated in Fig. 2b. The orientations of the uniform magnetic field, the velocity of the orthogonally moving fluid, and the generated Hall field are depicted in Fig. 1e. The field distribution along the cell axis, the *x-axis*, is shown in Fig. 2d.a. As evident, ${\text{H}}$ is uniform within the cylindrical space spanned by the electromagnet’s circular poles. However, away from the pole borders, ${\text{H}}$ is non-uniform and decays with a gradient, as shown in Fig. 2d.a-b.

To increase the non-uniformity of the field, we introduced a multipole magnetic field configuration for a few experiments. This configuration involved four laterally-magnetized bar magnets positioned around a cylinder with a 50 mm diameter, as seen in Fig. 2a,c. When used, this unit was inserted at the left entrance of the cell, with the long axis of each bar aligned along the direction of flow. At the lateral surface of each bar, ${\text{H}}$ is approximately 4.5 kOe, while ${\text{H}} \,=\, 0$ at the axis of the supporting cylinder. This setup creates a relatively strong $\nabla {\text{B}}$ near the inner surface of the tubing. It is worth noting that with an appropriate combination of uniform ${\text{B}}$ and $\nabla {\text{B}}$, we can simulate the field distribution of commercial permanent-magnet-type treatment devices, as classified by Gruber and Carda^76^. In addition, this setup is similar to those reported in Refs. ^9,22,23,27–32^ and can be used to simulate the action of “commercial magnetic treatment devices”^5,9,12,77^.

The cell casing is constructed from an insulating polycarbonate tube, denoted as I-case, and shown in Fig. 2e. To simulate a metallic tube (M-case), we inserted a stainless steel (SS) tube within the uniform-field space, as represented in Fig. 2f. We verified that the magnetic flux penetrates completely and uniformly through the interior space of the SS tube and the corresponding volume of the cell.

The stoichiometric amounts of ${\text{Ca}^{2+}}$-containing and ${\text{CO}_3^{2-}}$-containing compounds were dissolved in appropriate volumes of distilled water ($\sigma \approx 2 \mu$ S/cm) and placed in the Feed and Solution tanks, respectively. Each of ${\text{NaHCO}}_{3}$, ${\text{Na}_{2}\text{CO}}_{3}$, ${{\text{CaCl}}_{2}}$, and NaCl is considered to be completely dissociated; i.e., $\text{a}_{\text{ap}} \ll {\text{K}}_{\text{sp}}^{\text{eq}}$. For the sparingly soluble ${\text{CaCO}}_{3}$, supersaturation implies $\text{a}_{\text{ap}} \gg {\text{K}}_{\text{sp}}^{\text{eq}}$.

Each experiment was initiated by first circulating the ${\text{CO}}_3^{2-}$-containing solution with a mechanical pump at a rate of $56 \pm 2$ L/min. Before adding ${\text{CaCl}}_{2\mathrm{(aq)}}$, this circulation continued for about 10 minutes to ensure a stationary state, where all fluid in the Solution tank is equally exposed to the same cell conditions. After this period, ${\text{CaCl}}_{2\mathrm{(aq)}}$ was added via a computer-controlled peristaltic pump, with the rate and duration adjusted to deliver the exact amount of ${{\text{CaCl}}_{2}}$ needed to balance the reactions described in Eqs. (3)–(4); see the light blue area in, e.g., Figs. 3, 4.

Most experiments involved adding one liter of dissolved ${\text{CaCl}}_{2\mathrm{(aq)}}$ at a rate of 41.7 ml/min to twelve liters of ${\text{CO}}_3^{2-}$-containing solution, resulting in a final thirteen-liter solution with 7.306 mM of ${\text{Ca}}^{2+}$.

The rapid circulation of the fluid within the Solution tank, coupled with the slow addition from the Feed tank, helps (i) maintain concentration homogeneity throughout the experiment and (ii) ensure that the fluid in the Solution tank is consistently exposed to the controlled conditions of the cell environment (particularly the magnetic exposure). For experimental convenience, we used the slow addition of ${\text{CaCl2}}_{\mathrm{(aq)}}$. However, our findings and conclusions are expected to hold for other methods of mixing these incompatible waters.

Once the addition of ${\text{CaCl}}_{2\mathrm{(aq)}}$ is completed, the experiment continues for one more hour. During this period, the following conditions are maintained: constant mechanical agitation (500 rpm for the solution, 300 rpm for the feed), consistent circulation rate of the solutions, steady magnetic field value, the same cell casing, and in-situ real-time data acquisition of the experimental parameters.

Immediately after each experiment, with specified conditions such as ${\text{H}}$, Case, and the reactions of Eqs. (3) and (4), the whitish, turbid saline solution is filtered using an 8 $\mu$m filter paper. The residue is denoted as ${\text {Res}}_{8\mu {\text {m}}}^{\text {React}}$(H, Case), while the filtrate is labeled $\text{Filt}_{8\mu {\text{m}}}^{\text{React}}$, where React $\equiv$ reaction of Eq. (3) (the bicarbonate route) or Eq. (4) (the carbonate route).

Next, $\text{Filt}_{8\mu {\text{m}}}^{\text{React}}$ are filtered using a 0.22 $\mu$m filter. The residue is labeled ${\text {Res}}_{0.22\mu \text {m}}^{\text {React}}$(H, Case), and the filtrate is labeled $\text{Filt}_{0.22\mu {\text{m}}}^{\text{React}}$. Finally, one liter of $\text{Filt}_{0.22\mu {\text{m}}}^{\text{React}}$ is subjected to slow evaporation overnight, and the residue is denoted as ${\text {Res}}_{\text {evap}}^{\text {React}}$(H, Case).

The separation into three residues allows for a rough probing of the influence of ${\text{H}}$ across different particle size distributions. Although size distribution is typically analyzed by sophisticated techniques such as an optical particle counter^74^, here the ex-situ analysis, albeit crudely, examines the properties across these three ranges: ${\text {Res}}_{8\mu \text {m}}^{\text {React}}$(H, Case), ${\text {Res}}_{0.22\mu \text {m}}^{\text {React}}$(H, Case), and ${\text {Res}}_{\text {evap}}^{\text {React}}$(H, Case).

Various experimental, monitoring and characterizing, techniques were utilized in this study. The conductivity and pH probes from Atlas Scientific™  were interfaced with an Arduino™  microcontroller to provide real-time data analysis. These probes, calibrated before each series of runs, were positioned at various points along the setup shown in Fig. 2a.

Structural analysis was performed using a Cu ${\text{K}}_\alpha$ X-ray powder diffractometer (Miniflex from Siemens™). The obtained room-temperature diffractograms were refined using the Rietveld method as implemented by the FullProf suite software package developed by J. Rodriguez-Carvajal^78^. The structural parameters (space group, atomic positions, occupation, and thermal factors) for the calcite, vaterite, and aragonite phases were taken from Refs.^64–66^, respectively.

The mass magnetization $\mathrm{M(H, T)}$ and mass DC susceptibility, $\chi \mathrm{(T, H)} \,=\, \mathrm{M / H}$, of the obtained powdered residues were measured using the Physical Property Measurement System-Dynacool of Quantum Design™, operating within the ranges $2< {\text{T}} < 350$ K and $-140< {\text{H}} < 140$ kOe. The typical procedure involved: (i) a magnetization isotherm, ${\text{M}}(|{\text{H}}| < 140$ kOe, 300 K); (ii) an isofield magnetization, ${\text{M}}({\text{H}} \,=\, 10$ kOe, ${\text{T}}$), while cooling down to 2.5 K; (iii) a magnetization isotherm, ${\text{M}}(|{\text{H}}| < 140$ kOe, 2.5 K); and (iv) an isofield magnetization, ${\text{M}}({\text{H}} \,=\, 10$ kOe, ${\text{T}}$), while warming up to 300 K. Before each experiment, demagnetization with an oscillating field below 1 kOe was performed to eliminate possible residual fields.

Microstructure and elemental analysis were conducted using a scanning electron microscope (SEM) coupled with energy dispersive spectroscopy (EDS, model QUANTA™  FEG 250). We used a secondary electron detector, an acceleration voltage of 20 kV, a dwell time of 10 to 30 $\mu$s, and magnification ranging from 1000x to 5000x. The EDS data were processed using Espirit 1.9™  software which is provided with the setup. Samples were adhered to the sample holder with double-face carbon tape and coated with gold using a LEICA™  EM ACE600 metallizer under a current of 50 mA for 2 minutes. Due to the qualitative nature of EDS and the background presence of carbon from the sample support and oxygen from the storage environment, the determination of carbon and oxygen concentrations is primarily qualitative. However, the analysis of calcium and any trace elements, particularly *3d* transition-metal cations, is reliable. Additionally, since all SEM analyses were conducted on the same *pulverized* samples used for magnetization studies, we did not map the size distribution.

## Supplementary Information

Supplementary Information.

## Data Availability

The data that support the findings of this study are available in the main text and supplementary materials. Additional data are available from the corresponding author upon request.
