# Supplementary material for: Investigating the Antiscale Magnetic Treatment Controversy: Insights from the Model Calcium Carbonate Scalant
Source: Sci Rep. 2025 Jan 27;15:3441. doi: 10.1038/s41598-024-82048-9 (PMC11772624; doi:10.1038/s41598-024-82048-9)
Supplement: Supplementary file 1 — Supplementary Information. [file 41598_2024_82048_MOESM1_ESM.pdf]

Supplementary Materials: Investigating the Antiscale Magnetic Treatment Controversy: Insights from the Model Calcium Carbonate Scalant

M. ElMassalami,[1,2] M. S. Teixeira,[1] and A. Elzubair[3]

[1] *Physics Institute, Federal University of Rio de Janeiro, Post Office Box 68528, 21945-972 Rio de Janeiro, Brazil*

[2] *corresponding author: massalam@if.ufrj.br*

[3] *Military Institute of Engineering, Praça General Tibúrcio 80, 22290-270, Urca, Rio de Janeiro, RJ, Brazil*

(Dated: December 20, 2024)

We provide brief remarks on the analytical techniques employed in this work. Additionally, we include one Table and two sets of figures: The Table includes the elemental composition of $\mathrm{Res}_{8\mu m}^{\mathrm{React}}(\mathrm{H}, \mathrm{Case})$, which represents the elemental composition of other residues analyzed in this study. The first set of figures, shown in Figs.SM1-SM10, presents the structural, magnetic, microstructural, and compositional properties of $\mathrm{Res}_{0.22\mu m}^{\mathrm{React}}(\mathrm{H, Case})$, as well as a few cases of $\mathrm{Res}_{\mathrm{evap}}^{\mathrm{React}}(\mathrm{H, Case})$. These figures are very similar to those of $\mathrm{Res}_{8\mu m}^{\mathrm{React}}(\mathrm{H, Case})$ provided in the main text. The second set of figures, shown in Figs.SM11-SM18, describes the in-situ and ex-situ analyses related to $\mathrm{Res}_{\mathrm{Filter/Evap}}^{\mathrm{React}}$(0,Case,less-contaminant) and $\mathrm{Res}_{\mathrm{Filter/Evap}}^{\mathrm{React}}$($8+\nabla$B,Case,less-contaminant). The figures in the first set will be discussed in §2.2, §2.3, and §2.4, while those in the second set will be discussed in §2.5 of the main text.

SM1. Conductivity and PH analysis

Conductivity can be employed to monitor the progress of chemical reactions,[1] as it reflects variations in ionic strength during the conversion of reactants into products. This measurement provides insights into the reaction kinetics, mechanism, and whether the reaction involves intermediates or multiple steps. Additionally, we are interested in probing if there is any field-dependent variation in these parameters.

On the other hand, pH is commonly used to monitor variations in $[H^+]$ or $[OH-]$ in reactions involving acid/base chemistry. The time evolution of pH in a system depends on several factors, including the initial concentrations of reactants, the nature of the chemical reaction, and the buffering capacity of the solution.[2]

In this study, we followed the reactions of Eqs.3-4 by measuring conductivity, as shown in Figs.3(a)-4(a-b), and pH, as shown in Figs.3(b)-4(c) (see also Fig.SM11).

SM2. XRD analysis

Directly after obtaining the residues following the procedures outlined in Methods, §5, we subjected samples of each $\mathrm{Res}_{8\mu m}^{\mathrm{React}}(\mathrm{H}, \mathrm{Case})$, $\mathrm{Res}_{0.22\mu m}^{\mathrm{React}}(\mathrm{H}, \mathrm{Case})$, and $\mathrm{Res}_{\mathrm{evap}}^{\mathrm{React}}(\mathrm{H}, \mathrm{Case})$ to structural analysis, as described in §5. Representative refined patterns are shown in Figs.5, 6 (also see Figs.SM1 - SM3, SM12- SM13, and Table 1).

Using Rietveld analysis, we determined the phase content (Table 1) with precision governed by background level dispersion. However, as discussed in §2.3, the appearance of a relatively strong ferromagnetic signal suggests the presence of contaminating nano-sized particulates. These particulates have an XRD fingerprint below the detection limit of our conventional X-ray diffractometer.

SM3. Magnetization analysis

Magnetization and DC susceptibility curves of representative residues are shown in Figs.7-10 (see also Figs.SM5-SM8,SM14-SM15).

Each magnetization isotherm generally consists of two contributions: a *negative-in-H* diamagnetic signal, primarily due to the $CaCO_3$ polymorphs, and a *positive-in-H* signal originating from magnetic contaminants. The isofield DC susceptibility, on the other hand, measures the thermal evolution of the magnetic response when subjected to H = 10kOe, represented by the dashed vertical line in the magnetization curves.[3] It is defined as $\chi(\mathrm{H, T, Res}_{\mathrm{Filt}}^{\mathrm{React}}(\mathrm{H, Case})) \equiv \frac{\mathrm{M}\left(\mathrm{H,T,Res}_{\mathrm{Filt}}^{\mathrm{React}}(\mathrm{H,Case})\right)}{10\mathrm{kOe}}$, where Filt=0.22 or 8 μm and React corresponds to the reaction in Eq.3 or Eq.4. We did not analyze the magnetic properties of $\mathrm{Res}_{\mathrm{evap}}^{\mathrm{React}}(\mathrm{H, Case})$.

We observed that the high-field *positive-in-H* magnetization is relatively strong compared to the diamagnetic component; nonetheless, it is much smaller than the magnetization typically encountered in traditional ferrofluids with a moderate volume percentage.[4] Such stark differences in the magnetism of the two "magnetic fluids" emphasize once more the differences in concentration, distribution, composition, and microstructure of their ferromagnetic-like contaminants.

SM4. SEM analysis

Generally, the precipitation of a specific polymorph from a supersaturated solution is influenced by factors such as temperature, activity product, pH, and the kinetics of nucleation or growth. Additionally, some morphologies can be assisted and stabilized by external templates. For the three anhydrous polymorphs

TABLE SM1. The elemental analysis of $\mathrm{Res}_{8\mu\mathrm{m}}^{\mathrm{Eq.3/Eq.4}}(\mathrm{H},\mathrm{Case},\mathrm{Contaminant})$ is presented in normalized mass percentages, with standard deviations provided in parentheses. To determine the stoichiometry and chemical formulas, each element's normalized mass percentage should be multiplied by the factor (20.0174/atomic-weight). The ideal atomic percentages and normalized mass are listed in the last two rows of the table. Normalized mass percentages of additional trace elements (indicated in brackets) are given in the notes. This table extends the data provided in Table 1 in the main text.

			major elements			trace elements							
Reaction	H [kOe]	Case	C [%]	O [%]	Ca [%]	Al [%]	Ti [%]	Cr [%]	Mn [%]	Fe [%]	Co [%]	Ni [%]	Cu [%]
19Eq.3	0	I	14.3(2.9)	41.0(8.4)	42.1(9.8)	0.00	0.00	0.02(2)	0.01(1)	0.06(6)	0.01(1)	0.09(9)	1.4(4)[1]
21Eq.3	8	I	12.0(1.7)	41.0(2.6)	46.4(4.1)	0.08(1)	0.02(2)	0.01(1)	0.02(2)	0.09(3)	0.02(2)	0.06(1)	0.10(7)[2]
23Eq.3	0	M	12.9(1.8)	39.8(5.0)	46.8(6.3)	0.09(7)	0.01(1)	0.02(2)	0.03(2)	0.08(2)	0.01(1)	0.08(4)	0.06(3)[3]
25Eq.3	8	M	15.45(7.4)	35.8(6.7)	48.4(8.0)	0.09(1)	0.01(1)	0.01(1)	0.01(1)	0.10(5)	0.00	0.14(8)	0.09(8)[4]
32Eq.3	0	I	16.5(1.8)	46.4(4.6)	36.5(8)	0.00	0.01(1)	0.02(2)	0.03(3)	0.09(3)	0.00	0.00	0.04(3)
26Eq.3	8+∇H	I	19.5(3.7)	39.7	40.0(2.3)	0.05(3)	0.07(4)	0.01(1)	0.00	0.11(2)	0.01(1)	0.13(5)	0.24(16)[5]
8Eq.4	0	I	20.7(4.3)	42.7(2.2)	36.3(3.8)	0.05(5)	0.02(1)	0.01(1)	0.01(1)	0.09(3)	0.02(2)	0.06(4)	0.03(3)
12Eq.4	8	I	26.9(7.4)	37.1(2.2)	32.2(6.5)	0.34(30)	0.01(1)	0.01(1)	0.01(1)	0.10(2)	0.00	0.12(4)	1.33(25)
14Eq.4	0	M	29.3(6.1)	42.5(1.9)	28.0(5.6)	0.02(2)	0.01(1)	0.00	0.01(1)	0.07(1)	0.01(1)	0.05(2)	0.02(2)
16Eq.4	8	M	35.6(18.8)	37.3(7.9)	26.4(12.2)	0.07(2)	0.01(1)	0.01(1)	0.00	0.07(2)	0.00	0.07(2)	0.44(6)[6]
30Eq.4	0	I	17.7(2.9)	41.1(2.3)	40.7(0.6)	0.00	0.05(2)	0.00	0.00	0.03(3)	0.03(3)	0.02(2)	0.05(5)
28Eq.4	8+∇H	I	28.3(4.6)	43.4(1.3)	27.9(3.5)	0.05(3)	0.02(1)	0.01(1)	0.01(1)	0.08(4)	0.01(1)	0.08(4)	0.02(2)[7]
Ideal norm. atom.			12.00	40.00	40.00	0.00	0.00	0.00	0.00	0.00	0.00	0.00	0.00
Ideal norm. mass			12.00	47.96	40.04	0.00	0.00	0.00	0.00	0.00	0.00	0.00	0.00

[1] [Zn] = 1.01(37)% . [2] [S] = 0.2%. [3] [Si] = 0.13%. [4] EDS spectrum shown in Fig.SM18(a).
[5] [Na] = 0.18%; EDS spectrum shown in Fig.SM18(c). [6] [F] = 0.23%; EDS spectrum shown in Fig.SM18(b).
[7] [Na] = 0.3% and [Si] = 0.02; EDS spectrum shown in Fig.SM18(d).

of $CaCO_3$, the rhombohedral calcite typically forms as cuboid structures; the orthorhombic aragonite forms in acicular shapes, which can be monocrystalline needle-like, bundles of these needle-like crystals, polycrystalline dumbbells, or spherical forms; while the hexagonal vaterite forms as spherulites, dendritic flower-like or cauliflower-like shapes, discs, or monocrystalline hexagonal plates.[5–9] It has been reported that the varied morphologies, even within the same polymorph, may represent different stages of growth rather than stable forms specific to certain growth conditions.[9]

In this work, we investigated the evolution of the polymorphism, morphology, and elemental composition of $\mathrm{Res}_{\mathrm{Filt}}^{\mathrm{React}}(\mathrm{H},\mathrm{Case})$ using SEM analysis, following the procedures described in §5. Representative SEM micrographs are provided for both $\mathrm{Res}_{8\mu\mathrm{m}}^{\mathrm{Eq.3/Eq.4}}$ and $\mathrm{Res}_{0.22\mu\mathrm{m}}^{\mathrm{Eq.3/Eq.4}}$. The EDS spectra and element stoichiometry are shown only for $\mathrm{Res}_{8\mu\mathrm{m}}^{\mathrm{Eq.3/Eq.4}}$, as they faithfully represent those of other residues, particularly $\mathrm{Res}_{0.22\mu\mathrm{m}}^{\mathrm{Eq.3/Eq.4}}$. No SEM analysis was conducted on $\mathrm{Res}_{\mathrm{evap}}^{\mathrm{React}}(\mathrm{H},\mathrm{Case})$.

[1] P. W. Atkins and J. De Paula, *Atkins physical chemistry* (Oxford university press, 2006), 8th ed.

[2] A. Korchef, in *Crystallization and Applications*, edited by Y. B. Smida and R. Marzouki (IntechOpen, 2020), chap. 2, and references cited therein.

[3] Applying a high applied magnetic field for probing the magnetic susceptibility of a superparamagnetic-like behavior is dictated by the need to amplify their relatively weak magnetic signal against the background of a diamagnetic matrix.

[4] S. Odenbach, *Magnetoviscous Effects in Ferrofluids* (Springer Berlin Heidelberg, Berlin, Heidelberg, 2002).

[5] J.-P. Andreassen, R. Beck, and M. Nergaard, Faraday Discussions **159**, 247 (2012).

[6] R. Beck and J.-P. Andreassen, Crystal Research and Technology **47**, 404 (2012).

[7] I. Sunagawa, *Crystals: Growth, morphology and perfection* (2005).

[8] K. Song, J.-H. Bang, S.-C. Chae, J. Kim, and S.-W. Lee, RSC advances **12**, 19340 (2022).

[9] Q. Hu, J. Zhang, H. Teng, and U. Becker, American Mineralogist **97**, 1437 (2012).

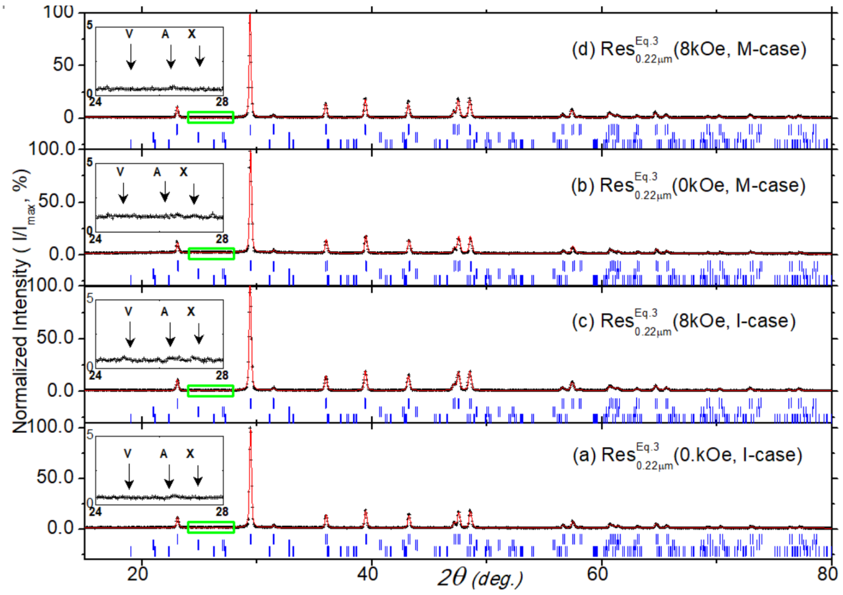

FIG. SM1. The analyzed diffractograms of $\mathrm{Res}^{\mathrm{Eq.3}}_{0.22\mu\mathrm{m}}$(H,Case) with **(a)** H=0 kOe and Case=I-case, **(b)** H=8.0 kOe and Case=I-case, **(c)** H=0 kOe and Case=M-case, and **(d)** H=8.0 kOe and Case=M-case. All intensities are normalized by the maximum intensity of the majority calcite phase, $\mathrm{I}_{\mathrm{max}}$. Each *Inset* is an expansion of the region wherein Bragg peaks of the minority phases (vatarite (V: (100)), aragonite (A: (111)), and their overlap, (X: V(101), A(102)) are expected to be manifested.

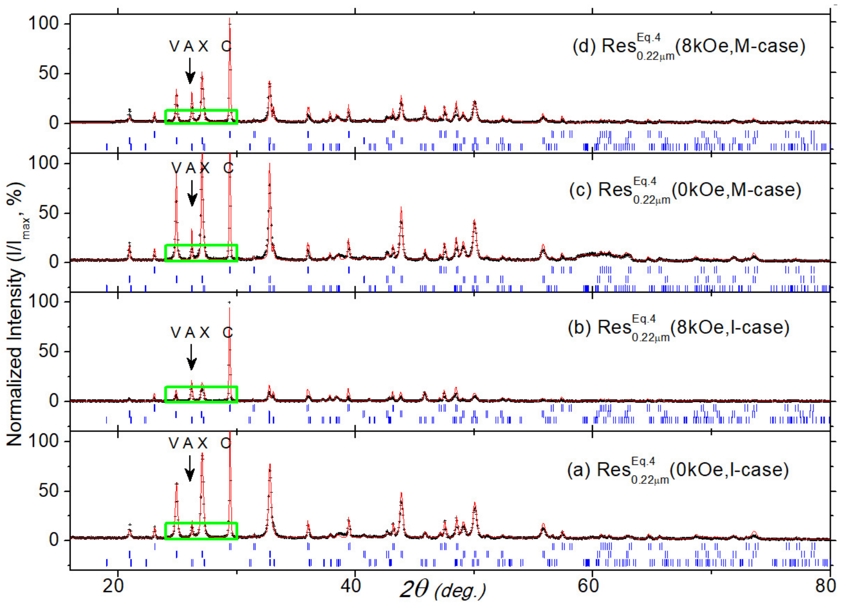

FIG. SM2. The analyzed diffractograms of $\mathrm{Res}^{\mathrm{Eq.4}}_{0.22\mu\mathrm{m}}$(H,Case) with **(a)** H=0 kOe and Case=I-case, **(b)** H=8.0 kOe and Case=I-case, **(c)** H=0 kOe and Case=M-case, and **(d)** H=8.0 kOe and Case=M-case. All intensities are normalized by the maximum intensity of the majority calcite phase, $\mathrm{I}_{\mathrm{max}}$. The *green rectangles* highlight the Bragg peaks of the calcite (C: (104)), vatarite (V:(100)), aragonite (A: (111)), or the overlap of aragonite and vatarite (X: V(101),A(102)).

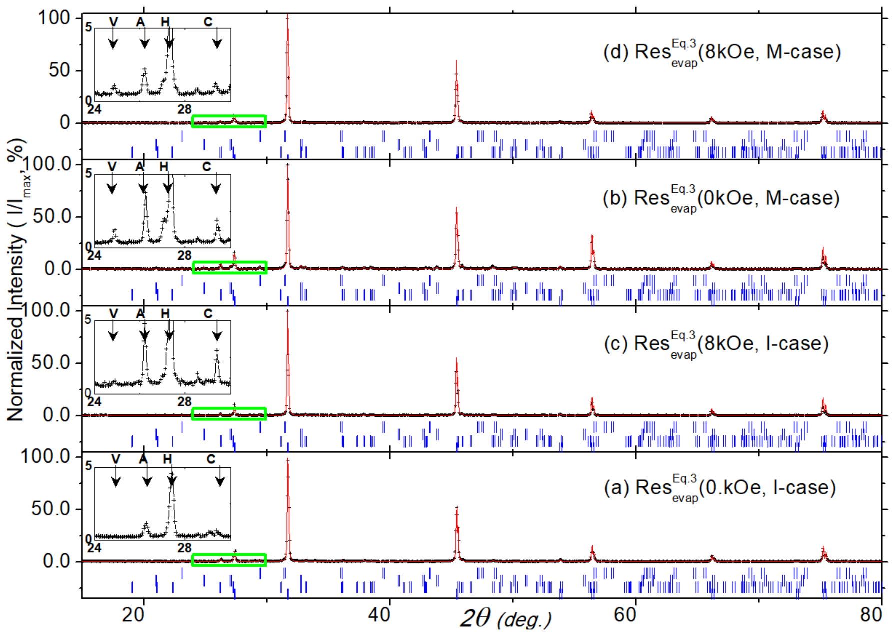

FIG. SM3. The refined patterns of $\mathrm{Res}^{\mathrm{Eq.3}}_{\mathrm{evap}}$(H,Case) with **(a)** H=0 kOe and Case=I-case, **(b)** H=8.0 kOe and Case=I-case, **(c)** H=0 kOe and Case=M-case, and **(d)** H=8.0 kOe and Case=M-case. All intensities are normalized by the maximum intensity of the majority halite phase, I_{max}. Each *Inset* is an expansion of the region showing Bragg peaks of the majority halite (H:(111)) and minority phases calcite (C: (104)), vatarite (V: (100)), aragonite (A: (111)), and their overlap, (X: V(101), A(102)).

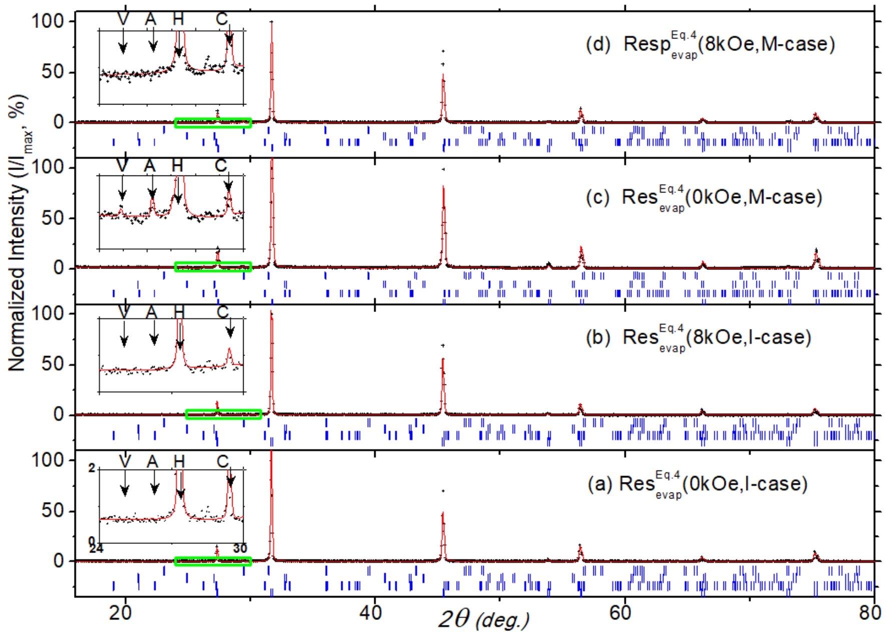

FIG. SM4. The refined patterns of $\mathrm{Res}^{\mathrm{Eq.4}}_{\mathrm{evap}}$(H,Case) with **(a)** H=0 kOe and Case=I-case, **(b)** H=8.0 kOe and Case=I-case, **(c)** H=0 kOe and Case=M-case, and **(d)** H=8.0 kOe and Case=M-case. All intensities are normalized by the maximum intensity of the majority halite phase, I_{max}. Each *Inset* is an expansion of the region showing Bragg peaks of the majority halite (H:(111)) and minority phases calcite (C: (104)), vatarite (V: (100)), aragonite (A: (111)), and their overlap, (X: V(101), A(102)).

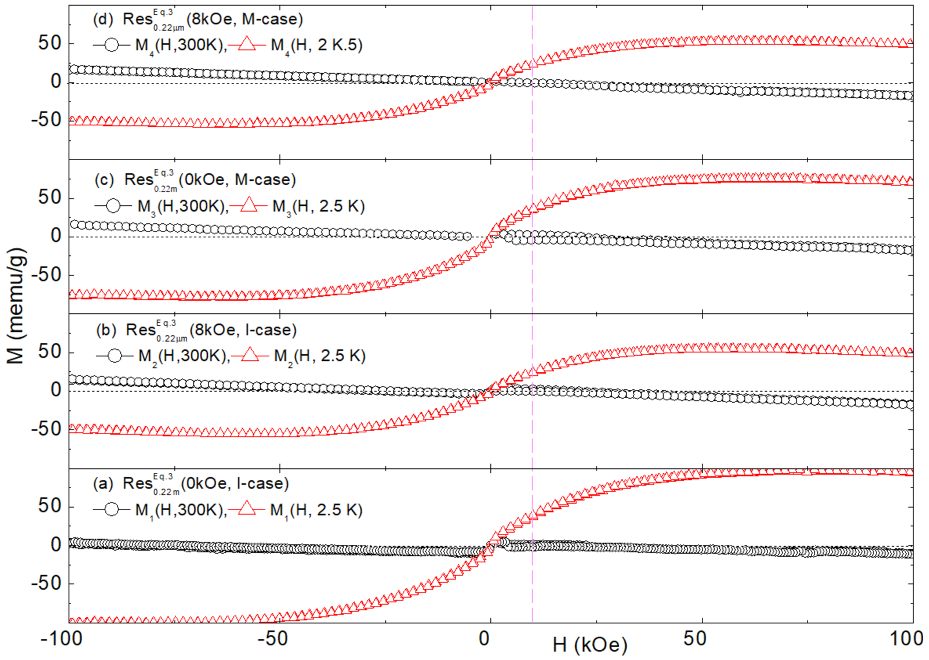

FIG. SM5. Magnetizations of $\mathrm{Res}^{\mathrm{Eq.3}}_{0.22\mu\mathrm{m}}$(H,Case): **(a)** M_1(H,T=300,2.5K) of $\mathrm{Res}^{\mathrm{Eq.3}}_{0.22\mu\mathrm{m}}$(0kOe,I-case). **(b)** M_2(H,T=300,2.5K) of $\mathrm{Res}^{\mathrm{Eq.3}}_{0.22\mu\mathrm{m}}$(8kOe,I-case). **(c)** M_3(H,T=300,2.5K) of $\mathrm{Res}^{\mathrm{Eq.3}}_{0.22\mu\mathrm{m}}$(0kOe,M-case). **(d)** M_4(H,T=300,2.5K) of $\mathrm{Res}^{\mathrm{Eq.3}}_{0.22\mu\mathrm{m}}$(8kOe,M-case).

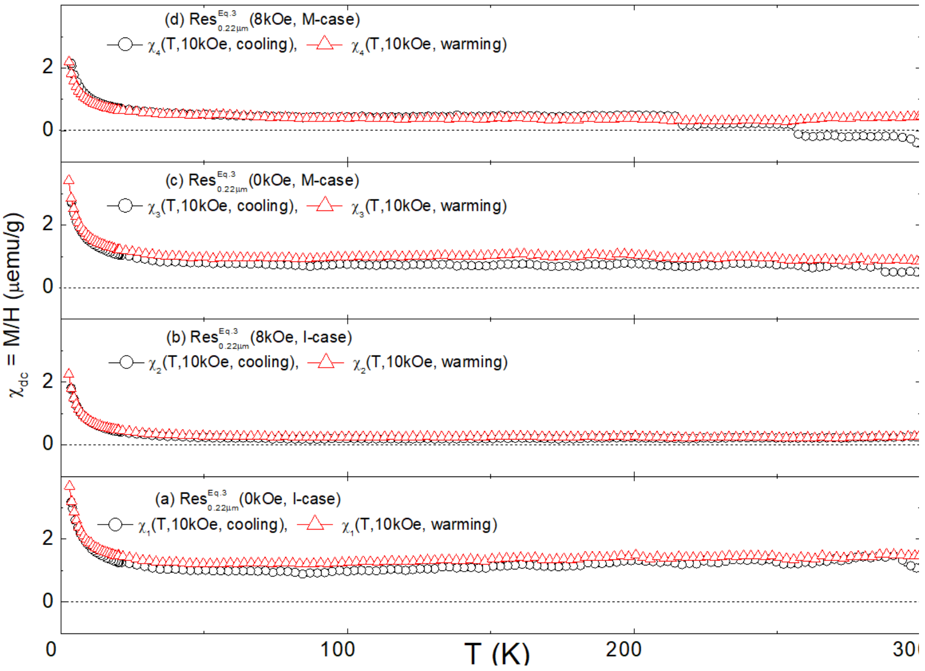

FIG. SM6. DC susceptibilities, χ_{dc}(T,10kOe)=M/H, of $\mathrm{Res}^{\mathrm{Eq.3}}_{0.22\mu\mathrm{m}}$(H,Case): **(a)** χ_1(T,10kOe,cooling/warming) of $\mathrm{Res}^{\mathrm{Eq.3}}_{0.22\mu\mathrm{m}}$(0kOe,I-case). **(b)** χ_2(T,10kOe,cooling/warming) of $\mathrm{Res}^{\mathrm{Eq.3}}_{0.22\mu\mathrm{m}}$(8kOe,I-case). **(c)** χ_3(T,10kOe,cooling/warming) of $\mathrm{Res}^{\mathrm{Eq.3}}_{0.22\mu\mathrm{m}}$(0kOe,M-case). **(d)** χ_4(T,10kOe,cooling/warming) of $\mathrm{Res}^{\mathrm{Eq.3}}_{0.22\mu\mathrm{m}}$(8kOe,M-case).

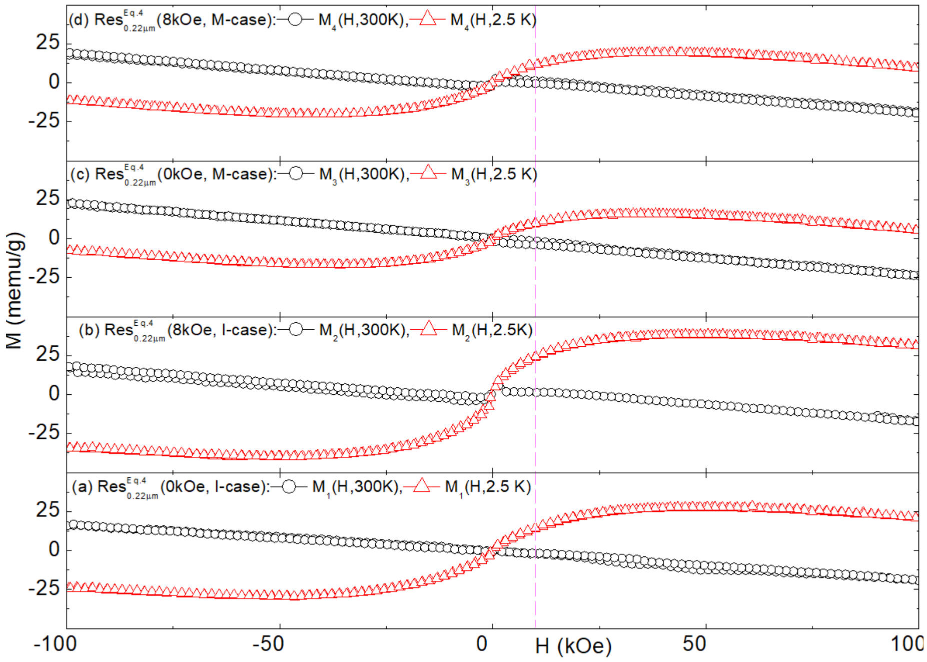

FIG. SM7. Magnetizations of $\mathrm{Res}^{\mathrm{Eq.4}}_{0.22\mu m}$(H,Case): **(a)** M_1(H,T=300,2.5K) of $\mathrm{Res}^{\mathrm{Eq.4}}_{0.22\mu m}$(0kOe,I-case). **(b)** M_2(H,T=300,2.5K) of $\mathrm{Res}^{\mathrm{Eq.4}}_{0.22\mu m}$(8kOe,I-case). **(c)** M_3(H,T=300,2.5K) of $\mathrm{Res}^{\mathrm{Eq.4}}_{0.22\mu m}$(0kOe,M-case). **(d)** M_4(H,T=300,2.5K) of $\mathrm{Res}^{\mathrm{Eq.4}}_{0.22\mu m}$(8kOe,M-case).

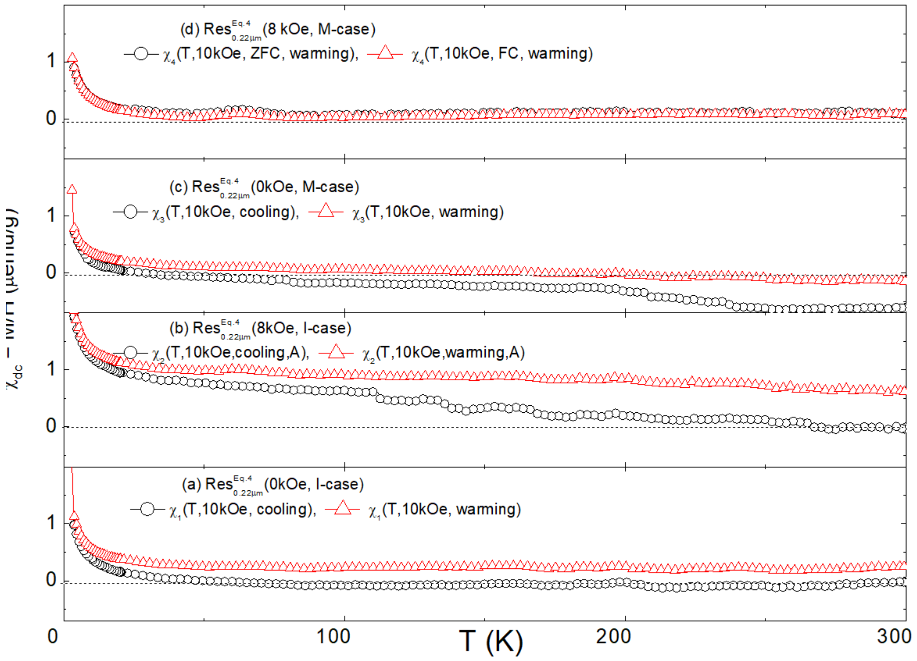

FIG. SM8. DC susceptibilities, χ_{dc}(T,10kOe)=M/H, of $\mathrm{Res}^{\mathrm{Eq.4}}_{0.22\mu m}$(H,Case): **(a)** χ_1(T,10kOe,cooling/warming) of $\mathrm{Res}^{\mathrm{Eq.4}}_{0.22\mu m}$(0kOe,I-case). **(b)** χ_2(T,10kOe,cooling/warming) of $\mathrm{Res}^{\mathrm{Eq.4}}_{0.22\mu m}$(8kOe,I-case). **(c)** χ_3(T,10kOe,cooling/warming) of $\mathrm{Res}^{\mathrm{Eq.4}}_{0.22\mu m}$(0kOe,M-case). **(d)** χ_4(T,10kOe,cooling/warming) of $\mathrm{Res}^{\mathrm{Eq.4}}_{0.22\mu m}$(8kOe,M-case).

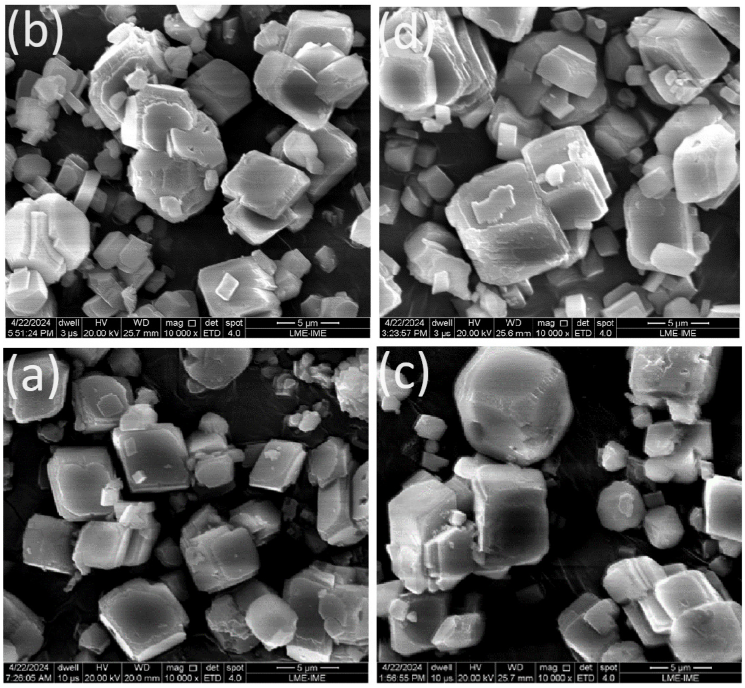

FIG. SM9. Morphologies and polymorphism of $\mathrm{Res}^{\mathrm{Eq.3}}_{0.22\mu\mathrm{m}}$(H,Case) when varying the applied magnetic field and cell casing: **(a)** SEM micrographs of $\mathrm{Res}^{\mathrm{Eq.3}}_{0.22\mu\mathrm{m}}$(0kOe,I-case). **(b)** SEM micrographs of $\mathrm{Res}^{\mathrm{Eq.3}}_{0.22\mu\mathrm{m}}$(8kOe,I-case). **(c)** SEM micrographs of $\mathrm{Res}^{\mathrm{Eq.3}}_{0.22\mu\mathrm{m}}$(0kOe,M-case). **(d)** SEM micrographs of $\mathrm{Res}^{\mathrm{Eq.3}}_{0.22\mu\mathrm{m}}$(8kOe,M-case).

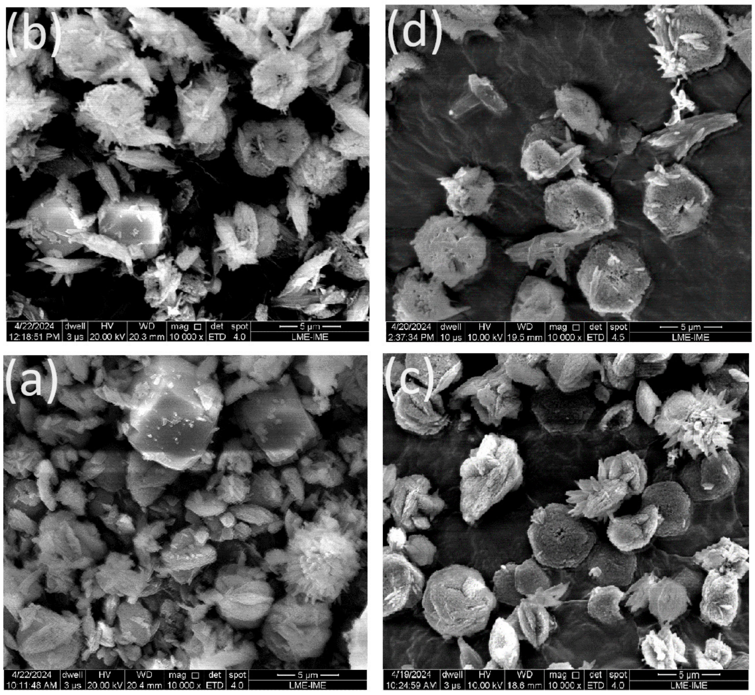

FIG. SM10. Morphologies and polymorphism of $\mathrm{Res}^{\mathrm{Eq.4}}_{0.22\mu\mathrm{m}}$(H,Case) when varying the applied magnetic field and cell casing: **(a)** SEM micrographs of $\mathrm{Res}^{\mathrm{Eq.4}}_{0.22\mu\mathrm{m}}$(0kOe,I-case). **(b)** SEM micrographs of $\mathrm{Res}^{\mathrm{Eq.4}}_{0.22\mu\mathrm{m}}$(8kOe,I-case). **(c)** SEM micrographs of $\mathrm{Res}^{\mathrm{Eq.4}}_{0.22\mu\mathrm{m}}$(0kOe,M-case). **(d)** SEM micrographs of $\mathrm{Res}^{\mathrm{Eq.4}}_{0.22\mu\mathrm{m}}$(8kOe,M-case).

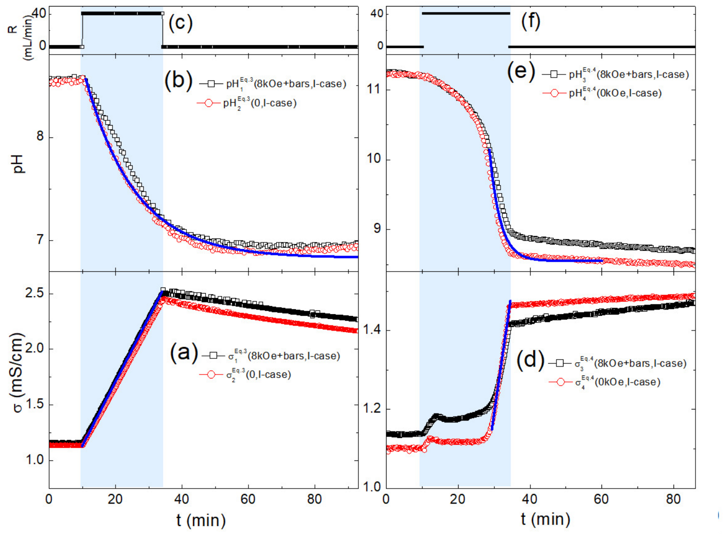

FIG. SM11. Evolution of conductivity and pH of both reactions (Eq.3,4) when reducing the corrosion-related impurities and adding an additional non-uniform field, ∇B. **(a)** Evolution of $\sigma_i^{\mathrm{Eq.3}}$(H=0.0,8.0kOe+∇B,I-case). **(b)** Evolution of $\mathrm{pH}_i^{\mathrm{Eq.3}}$(H=0.0,8.0kOe+∇B,I-case). **(c)** Evolution of the rate of controlled addition of $CaCl_{2(aq)}$ to the circulating $NaHCO_{3(aq)}$ solution. **(d)** Evolution of $\sigma_i^{\mathrm{Eq.4}}$(H=0.0,8.0kOe+∇B,I-case). **(e)** Evolution of $\mathrm{pH}_i^{\mathrm{Eq.4}}$(H=0.0,8.0kOe+∇B,I-case). The thick blue lines describe the linear evolution of conductivities while the solid blue curves simulate the exponentially decaying function of Eq.7: While the decay in pH of *panel (b)* starts directly after introducing $CaCl_{2(aq)}$, the sharp decay in pH of *panel (e)* start around 15 minutes later, after an additional weaker decay, attributed to formation of vaterite phase (see text). **(f)** Evolution of the rate of controlled addition of $CaCl_{2(aq)}$ to the circulating $Na_2CO_{3(aq)}$ solution. The light-blue area highlights the onset and duration of $CaCl_{2(aq)}$ addition.

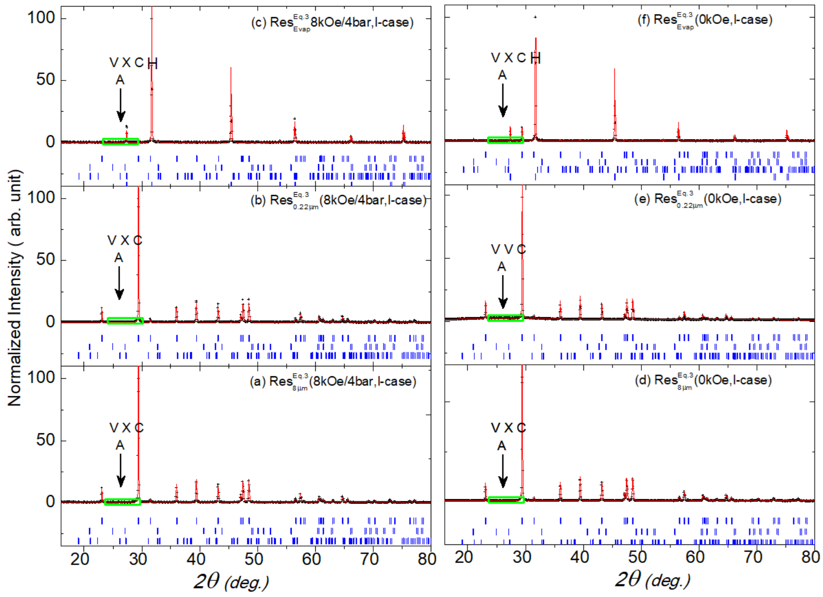

FIG. SM12. The refined patterns of $\mathrm{Res}_{\mathrm{Filter/evap}}^{\mathrm{Eq.3}}$(H,I-case) when reducing the corrosion-related impurities [∇B denotes the additional non-uniform contribution introduced by the four bar magnets (4bar)]: **(a)** H=8.0 kOe+∇B, Filter=8μm, Case=I-case, **(b)** H=8.0 kOe+∇B, Filter=0.22μm, Case=I-case, **(c)** H=8.0 kOe+∇B, Evaporation, Case=I-case, **(d)** H=0 kOe, Filter=8μm, Case=I-case, **(e)** H=0 kOe, Filter=0.22μm, Case=I-case, **(f)** H=0 kOe, Evaporation, Case=I-case, The green rectangle emphasizes that any minority phase must be within the background statistics. All intensities are normalized by the maximum intensity of the majority phase, I_{max}.

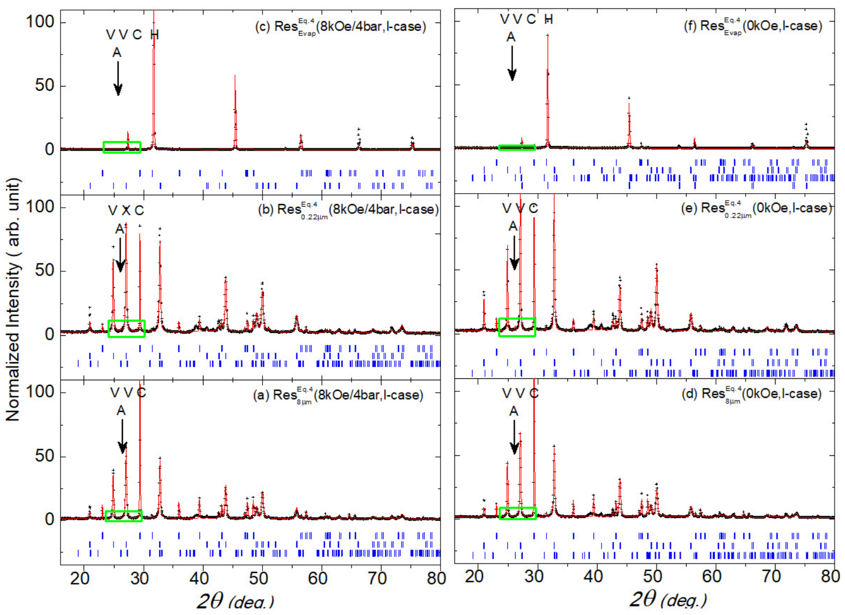

FIG. SM13. The refined patterns of $\mathrm{Res}^{\mathrm{Eq.4}}_{\mathrm{Filter/evap}}$(H,I-case) after using the cell configuration of Fig.2 with following conditions [∇B denotes the additional non-uniform contribution introduced by the four bar magnets (4bar)]: **(a)** H=8.0 kOe+∇B, Filter=8μm, Case=I-case, **(b)** H=8.0 kOe+∇B, Filter=0.22μm, Case=I-case, **(c)** H=8.0 kOe+∇B, Evaporation, Case=I-case, **(d)** H=0 kOe, Filter=8μm, Case=I-case, **(e)** H=0 kOe, Filter=0.22μm, Case=I-case, **(f)** H=0 kOe, Evaporation, Case=I-case. The *green rectangles* highlight the Bragg peaks of the minority phase (see text). All intensities are normalized by the maximum intensity of the majority phase, I_{max}.

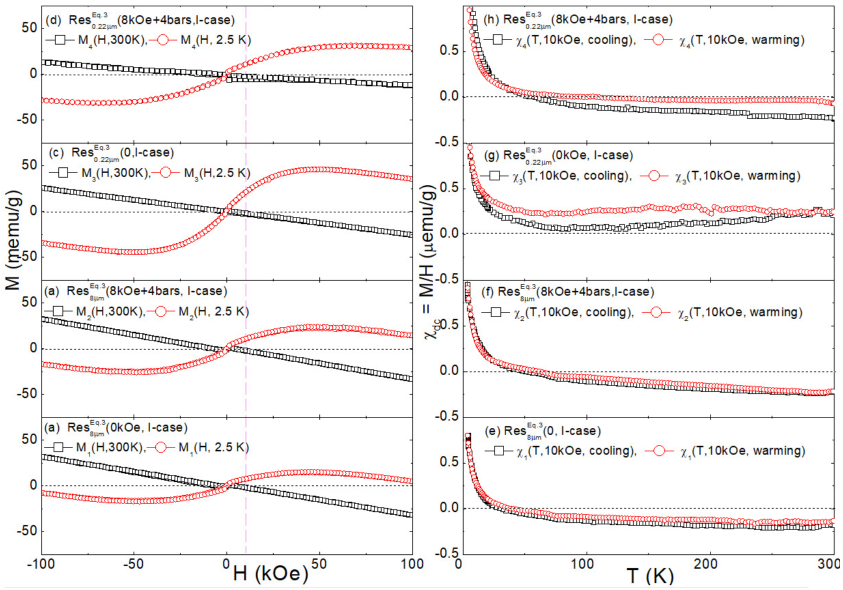

FIG. SM14. Magnetization of $\mathrm{Res}^{\mathrm{Eq.3}}_{\mathrm{Filt}}$(H,Case,less-contaminant): **(a)** M_1(H,T=300,2.5K) of $\mathrm{Res}^{\mathrm{Eq.3}}_{8\mu\mathrm{m}}$(0kOe,I-case), **(b)** M_2(H,T=300,2.5K) of $\mathrm{Res}^{\mathrm{Eq.3}}_{8\mu\mathrm{m}}$(8kOe+$\nabla$B,I-case), **(c)** M_3(H,T=300,2.5K) of $\mathrm{Res}^{\mathrm{Eq.3}}_{0.22\mu\mathrm{m}}$(0kOe,I-case). **(d)** M_4(H,T=300,2.5K) of $\mathrm{Res}^{\mathrm{Eq.3}}_{0.22\mu\mathrm{m}}$(8kOe+$\nabla$B,I-case), DC susceptibilities, χ_{dc}(T,10kOe)=M/H, of $\mathrm{Res}^{\mathrm{Eq.3}}_{\mathrm{Filt}}$(H,Case): **(e)** χ_1(T,10kOe,cooling/warming) of $\mathrm{Res}^{\mathrm{Eq.3}}_{8\mu\mathrm{m}}$(0kOe,I-case), **(f)** χ_2(T,10kOe,cooling/warming) of $\mathrm{Res}^{\mathrm{Eq.3}}_{8\mu\mathrm{m}}$(8kOe+$\nabla$B,I-case): **(g)** χ_3(T,10kOe,cooling/warming) of $\mathrm{Res}^{\mathrm{Eq.3}}_{0.22\mu\mathrm{m}}$(0kOe,I-case). **(h)** χ_4(T,10kOe,cooling/warming) of $\mathrm{Res}^{\mathrm{Eq.3}}_{0.22\mu\mathrm{m}}$(8kOe+$\nabla$B,I-case).

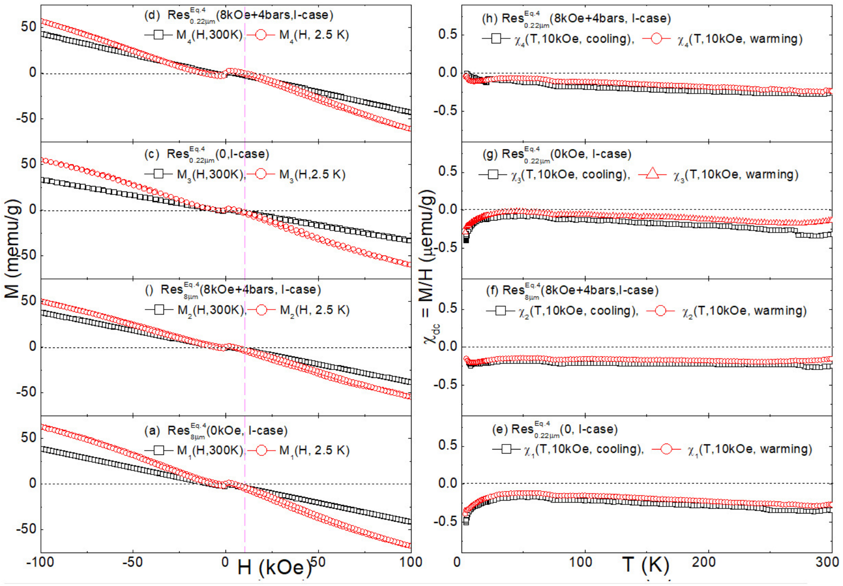

FIG. SM15. Magnetization of Res$^{Eq.4}_{8\mu m}$(H,Case): **(a)** M_1(H,T=300,2.5K) of Res$^{Eq.4}_{8\mu m}$(0kOe,I-case), **(b)** M_2(H,T=300,2.5K) of Res$^{Eq.4}_{8\mu m}$(8kOe+∇B,I-case), **(c)** M_3(H,T=300,2.5K) of Res$^{Eq.4}_{0.22\mu m}$(0kOe,I-case), **(d)** M_4(H,T=300,2.5K) of Res$^{Eq4}_{0.22\mu m}$(8kOe+∇B,I-case). DC susceptibilities. χ_{dc}(T,10kOe)=M/H, of Res$^{Eq.4}_{8\mu m}$(H,Case): **(e)** χ_1(T,10kOe,cooling/warming) of Res$^{Eq.4}_{8\mu m}$(0kOe,I-case), **(f)** χ_2(T,10kOe,cooling/warming) of Res$^{Eq.4}_{8\mu m}$(8kOe+∇B,I-case), **(g)** χ_3(T,10kOe,cooling/warming) of Res$^{Eq.4}_{0.22\mu m}$(0kOe,I-case), **(h)** χ_4(T,10kOe,cooling/warming) of Res$^{Eq.4}_{0.22\mu m}$(8kOe+∇B,I-case).

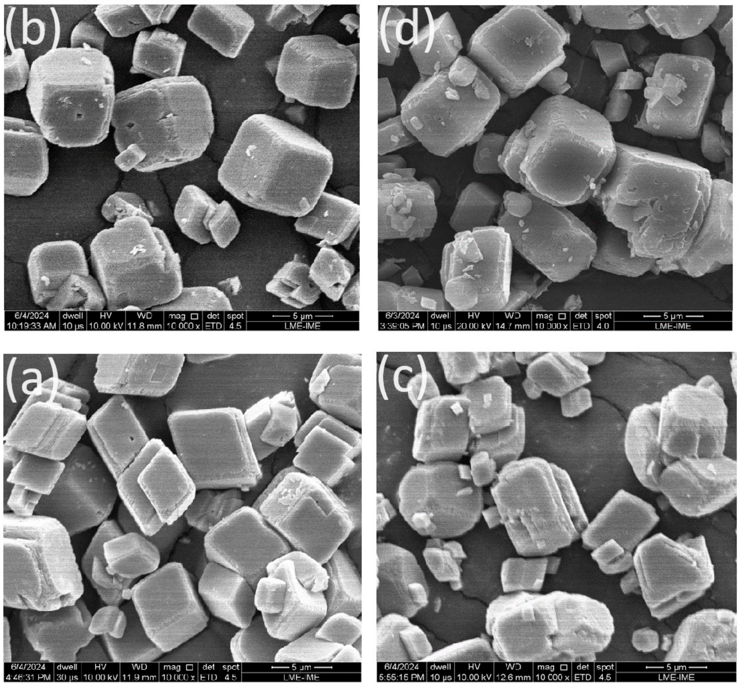

FIG. SM16. Morphologies and polymorphism of Res$^{Eq.3}_{Filt}$(H,Case,less-contaminant) when varying the applied magnetic field and the Filter: **(a)** SEM micrographs of Res$^{Eq.3}_{8\mu m}$(0kOe,I-case,less-contaminant), **(b)** SEM micrographs of Res$^{Eq.3}_{8\mu m}$(8kOe+∇B,I-case,less-contaminant), **(c)** SEM micrographs of Res$^{Eq.3}_{0.22\mu m}$(0kOe,I-case,less-contaminant), **(d)** SEM micrographs of Res$^{Eq.3}_{0.22\mu m}$(8kOe+∇B,I-case,less-contaminant). It is recalled that all samples were pulverized; therefore, the granular dimensions observed in these micrographs are not significant.

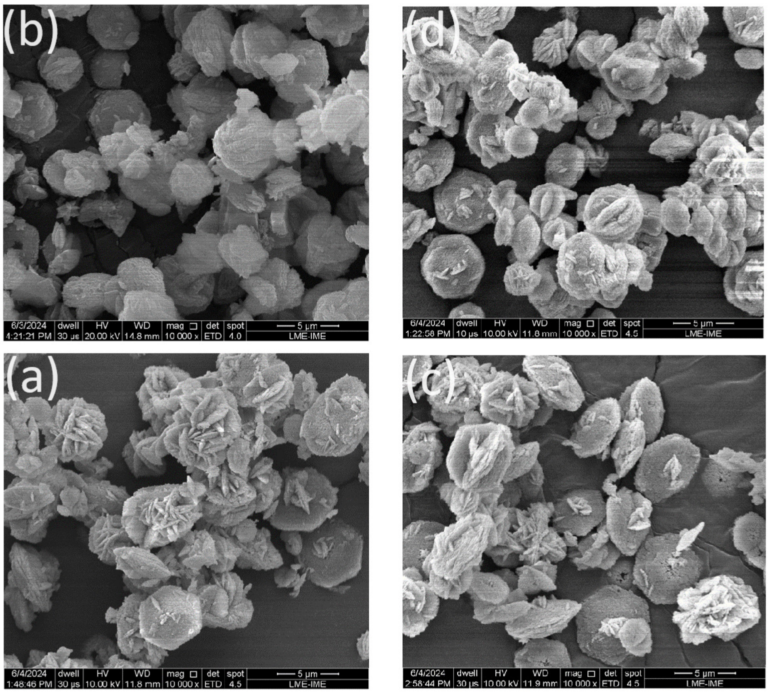

FIG. SM17. Morphologies and polymorphism of $\text{Res}^{\text{Eq.4}}_{\text{Filt}}$(H,Case,less-contaminant) when varying the applied magnetic field and the Filter: **(a)** SEM micrographs of $\text{Res}^{\text{Eq.4}}_{8\mu\text{m}}$(0kOe,I-case,less-contaminant), **(b)** SEM micrographs of $\text{Res}^{\text{Eq.4}}_{8\mu\text{m}}$(8kOe+$\nabla$B,I-case,less-contaminant), **(c)** SEM micrographs of $\text{Res}^{\text{Eq.4}}_{0.22\mu\text{m}}$(0kOe,I-case,less-contaminant), **(d)** SEM micrographs of $\text{Res}^{\text{Eq.4}}_{0.22\mu\text{m}}$(8kOe+$\nabla$B,I-case,less-contaminant). All samples were pulverized; therefore, the granular dimensions observed in these micrographs are not significant.

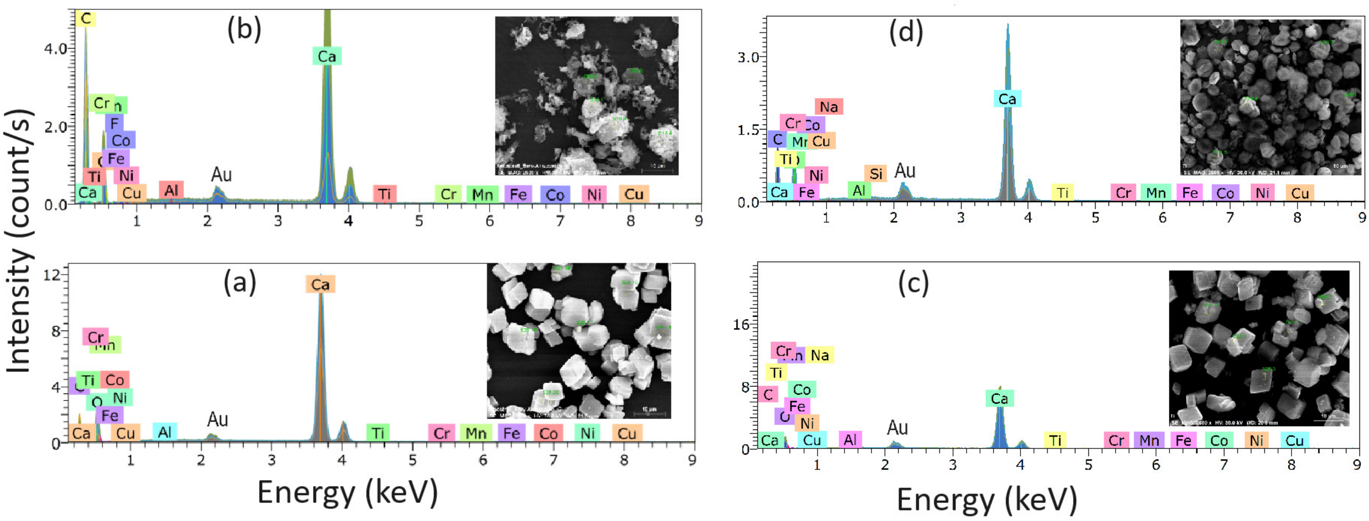

FIG. SM18. EDS spectrum and SEM micrographs of four representative samples: **(a)** EDS spectrum of $\text{Res}^{\text{Eq.3}}_{8\mu m}(8\text{kOe, M-case})$. **(b)** EDS spectrum of $\text{Res}^{\text{Eq.4}}_{8\mu m}(8\text{kOe, M-case})$. **(c)** EDS spectrum of $\text{Res}^{\text{Eq.3}}_{8\mu m}(8\text{kOe} + \nabla\text{B, I-case, less} - \text{contaminant})$. **(d)** EDS spectrum of $\text{Res}^{\text{Eq.4}}_{8\mu m}(8\text{kOe} + \nabla\text{B, I-case, less} - \text{contaminant})$. *Insets*: SEM micrograph of the crossponding residue, similar to the ones shown in Figs.11(d), 12(d), SM16(b), SM17(b), respectively. EDS spectra are taken at spots indicated by corresponding green symbols in the corresponding SEM micrographs. The derived normalized mass percentages calculated from such spectra are shown in Table SM1. Atomic labels indicate the energy of the K-series for each element. Au is related to the coating gold.
